# Mathematical model on Alzheimer’s disease

**DOI:** 10.1186/s12918-016-0348-2

**Published:** 2016-11-18

**Authors:** Wenrui Hao, Avner Friedman

**Affiliations:** 1 Department of Mathematics, The Penn State University, University Park, 16802 PA USA; 2 Mathematical Biosciences Institute & Department of Mathematics, The Ohio State University, Columbus, 43210 OH USA

**Keywords:** Alzheimer disease, Mathematical modeling, Drug treatment

## Abstract

**Background:**

Alzheimer disease (AD) is a progressive neurodegenerative disease that destroys memory and cognitive skills. AD is characterized by the presence of two types of neuropathological hallmarks: extracellular plaques consisting of amyloid *β*-peptides and intracellular neurofibrillary tangles of hyperphosphorylated tau proteins. The disease affects 5 million people in the United States and 44 million world-wide. Currently there is no drug that can cure, stop or even slow the progression of the disease. If no cure is found, by 2050 the number of alzheimer’s patients in the U.S. will reach 15 million and the cost of caring for them will exceed $ 1 trillion annually.

**Results:**

The present paper develops a mathematical model of AD that includes neurons, astrocytes, microglias and peripheral macrophages, as well as amyloid *β* aggregation and hyperphosphorylated tau proteins. The model is represented by a system of partial differential equations. The model is used to simulate the effect of drugs that either failed in clinical trials, or are currently in clinical trials.

**Conclusions:**

Based on these simulations it is suggested that combined therapy with TNF- *α* inhibitor and anti amyloid *β* could yield significant efficacy in slowing the progression of AD.

## Background

AD is the most common form of dementia. The disease is an irreversible, progressive, brain disorder that destroys memory and cognitive skills, and eventually the ability to carry out even the simplest tasks. While the genetic inheritability of AD is in the range of 50 –80% [1, 2], the cause of the disease is mostly unknown. The disease strikes ageing people typically 65 or older, and twice more women than men. In 2015 there were more than 5 million people in the United States with AD, and 44 millions world-wide [3]. The cost of caring for AD patients in the U.S. was estimated at $226 billions for 2015 [3].

AD is characterized by the presence of two types of neuropathological hallmarks: extracellular plaques and intracellular neurofibrillary tangles (NFTs). The extracellular plaques consist primarily of amyloid *β*-peptide (A *β*) deposits. The NFTs are intraneural aggregation of hyperphosphorylated tau proteins. Reactive oxygen species (ROS) appears to be one of the early events in the progression of the disease [4]. Amyloid precursor protein (APP) on neurons membrane constitutively shed A *β* peptides [5]. High levels of ROS promote abnormal deposition of A *β* [4, 6]. Tau protein in the central nervous system (CNS) is predominantly expressed in neurons; its main role is to promote microtubles assembly and stability. Glycogen synthase kinase-type 3(GSK-3) is activated by the abnormally produced A *β*, and it mediates the hyperphosphorylation of tau proteins [4, 6–9].

The hyperphosphorylated tau proteins cause microtuble depolymerization and destruction, as they aggregate to form neurofibrillary tangles. This results in neuronal death and release of the NFTs to the extracellular environment [4, 10].

The non-neuronal cells in the brain consist of cells that support neurons directly, mostly astrocytes, and immune cells.

Microglias are the resident macrophages in the brain. They constitute the main active immune cells in the brain. They are activated by soluble A *β* oligomers which build up from the A *β* deposits [11, 12].

Astrocytes are in close proximity to neurons. They support neuronal cross-talk, and mediate the transport of nutrients from the blood to neurons. Astrocytes are activated primarily by TNF- *α*, but also by A *β* [10, 13–16]. Activated astrocytes produce A *β*, but at a smaller rate than neurons [16]. Activated astrocytes also produce MCP-1, which attracts monocytes from the blood into the plaques [17–19]. The monocytes differentiate into proinflammatory macrophages, $\hat {M}_{1}$, but may then change phenotype into anti-inflammatory $\hat {M}_{2}$ macrophage. Activated microglias have two phenotypes: proinflammatory *M*_1_ macroglia and anti-inflammatory *M*_2_ macroglia [12, 20]. Macrophages have a major role in A *β* clearance [12, 20], but activated microglia are poorly phagocytic for A *β* compared to peripheral macrophages [21]. *M*_1_ and $\hat {M}_{1}$ macrophages are neurotoxic; they produce proinflammatory cytokines TNF- *α*, IL-6, IL-12 and IL-1 *β* [20, 22, 23]. *M*_2_ microglias and peripheral $\hat {M}_{2}$ macrophages produce anti-inflammatory cytokines IL-10, IL-13, IL-4 and TGF- *β* [20]. The neuronal stress caused by the proinflammatory cytokines, is resisted by IL-10, IL-13 and IL-4, but nevertheless it contributes to neuronal damage and death [20, 22, 23].

There are currently no drugs that can cure AD, or stop its progression. Many clinical trials of drugs aimed at preventing or clearing the A *β* and tau pathology have failed to demonstrate efficacy [24–27]. Currently the only treatment of AD is by medications that are used to treat the symptoms of the disease.

The role of TGF- *β* is somewhat controversial [28]. On one hand, TGF- *β* provides protection against neuroninflammation and neurondegeneration [29–34], but on the other hand, TGF- *β*-induced TIAF1 interacts with amyloid fibrils to favorably support plaque formation [28], and blocking TGF- *β*-smad2/3 in peripheral macrophages mitigates AD pathology [35].

**Fig. 1:**
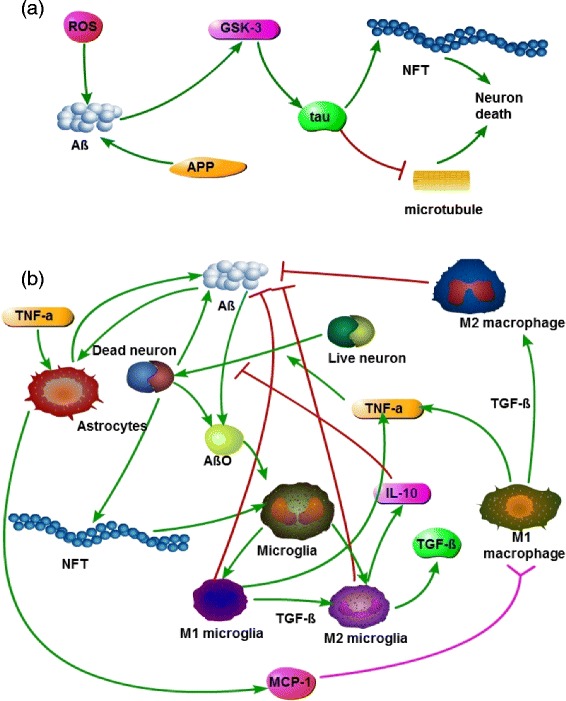
Schematic network in AD: **a** Amyloid precurser protein (APP) sheds Amyloid *β* peptides. ROS promotes abnormal production of A *β* [5, 6], which activates GSK-3 [4, 6, 8]. Activated GSK-3 mediates hyperphosphrylation of tau proteins [4, 6], which results in the formation of NFTs [10] and destruction of microtubules [4, 10], leading to neuron death. **b** Astrocytes are activated by $A_{\beta }^{o}$ [10, 16] and TNF- *α* [14, 15], and they produce MCP-1 [17–19], which attracts macrophages into the tissue [17, 19]. NFT activates microglias [10, 13, 15]. Activated proinflammatory microglias and microphages produce TNF- *α* and other proinflammatory cytokines [20, 22, 23], while anti-inflammatory microglias and macrophages produce IL-10 and other anti-inflammatory cytokines [20, 22, 23]. Dead neurons release A *β* and NFTs, and soluble A *β* oligomers activate microglia [11, 12]. Activated astrocytes secrete A *β* [16]. A *β* deposit is reduced through endocytosis by microglia and macrophages [12, 20]

Figure 1 is a schematics of the network associated with the progression of AD. Figure 1 a shows the network within a neuron which leads from ROS to NFTs and the destruction of microtubules. Figure 1 b shows the network of activated cells, microglia, astrocyte and monocyte-derived macrophages and their effect on neurons and their microenvironment.

In this paper we develop a mathematical model of AD. The model is represented by a system of partial differential equations (PDEs) based on Fig. 1. For simplicity we represent all the proinflammatory cytokines by TNF- *α*, and all the anti-inflammatory cytokines by IL-10.

We shall use our model to conduct in silico trials with several drugs: TNF- *α* inhibitor, anti-A *β* drug, MCP-1 inhibitor, and injection of TGF- *β*. Simulations of the model show that continuous treatment with TNF- *α* inhibitor yields a slight decrease the death of neurons, and anti-A *β* drug yields a slight decrease in the aggregation of A *β* over 10 years period, while the benefits from injection of TGF- *β* and MCP-1 inhibitor drugs are negligible. This suggests that clinical trials consider combination therapy with TNF- *α* and anti-A *β* drugs.

We note that Fig. 1 does not display neurites: the projections of axons and dendrites from the body of neurons. It is known that the aggregations of A *β* mediate rapid disruption of synaptic plasticity and memory [36–39]. Thus the progression of AD in terms of reduction in dendritic complexity and synaptic dysfunction will not be considered in the present paper.

We conclude the Introduction by mentioning earlier mathematical models which deal with some aspects of AD: A *β* polymerization [40], A *β* plaque formation and the role of prions interacting with A *β* [41, 42], linear cross-talk among brain cells and A *β* [43], and the influence of SORLA on AD progression [44, 45].

## Methods

### Mathematical model

#### Model’s variables

The mathematical model is based on Fig. 1 and is represented by a system of partial differential equations. Table 1 lists the variables used in the model.

**Table 1:** The variables of the model; concentration and densities are in units of *g*/*c* *m* ^3^ for cells and *g*/*m* *l* for cytokines

ROS (*R*):	Reactive oxygen species	GSK-3 (*G*):	Glycogen synthase kinase-type 3
$A_{\beta }^{i}$:	Amyloid *β* inside neurons	$A_{\beta }^{o}$:	Amyloid *β* outside neurons
NFT (*F*_*i*_):	Neuronfibrillary tangle inside neurons	NFT (*F*_*o*_):	Neuronfibrillary tangle outside neurons
APP (*A*_*P*_):	Amyloid precursor protein	A *β*O (*A*_*O*_):	Amyloid *β* oligomer (soluble)
TNF- *α* (*T*_*α*_):	Tumor necrosis factor alpha	TGF- *β* (*T*_*β*_):	Transforming growth factor beta
IL-10 (*I*_10_):	Interleukin 10	*P*:	MCP-1
*M*_1_:	Proinflammatory microglias	*M*_2_:	Anti-inflammatory microglias
MG (*M*_*G*_):	Microglias	N:	Live neurons
A :	Astrocytes	*N*_*d*_:	Dead neurons
$\hat {M}_{1}$	Peripheral proinflammatory macrophages	$\hat {M}_{2}$:	Peripheral anti-inflammatory macrophages
*τ*	hyperphosphorylated tau protein	H	High mobility group box 1 (HMGB1)

#### Equations for A *β*

The amyloid- *β* within neurons, $A^{i}_{\beta }$, are constitutively released from APP at a rate $\lambda _{\beta }^{i}$ and are degraded at a rate $d_{A_{\beta }^{i}}$. Under reactive oxidative stress, *R*, $A_{\beta }^{i}$ is overproduced. Hence the equation for $A_{\beta }^{i}$ is given by

1 $$\begin{array}{@{}rcl@{}} \frac{\partial A_{\beta}^{i}}{\partial t}&=&\left(\underbrace{\lambda_{\beta}^{i}(1+R)}_{production}\underbrace{-d_{A^{i}_{\beta}} A^{i}_{\beta}}_{degradation}\right)\frac{N}{N_{0}}, \end{array} $$

where *N*_0_ is the reference density of the neuron cells in the brain.

The extracellular amyloid- *β* peptides satisfy the following equation:

2 $$ \begin{aligned} &{}\frac{\partial A_{\beta}^{o}}{\partial t}=\underbrace{A_{\beta}^{i}\left|\frac{\partial N}{\partial t}\right|+\lambda_{N}\frac{N}{N_{0}}+\lambda_{A}\frac{A}{A_{0}}}_{production}\\ & \quad \underbrace{-\left(d_{A^{o}_{\beta} \hat{M}} \left(\hat{M}_{1}+\theta\hat{M}_{2}\right)+d_{A^{o}_{\beta} {M}}\left({M}_{1}+\theta{M}_{2}\right)\right)\frac{A_{\beta}^{o}}{A_{\beta}^{o}+\bar{K}_{A_{\beta}^{o}}}}_{clearance} \,, \end{aligned}  $$

where $\bar {K}_{A_{\beta }^{o}}$ is a Michaelis-Menten coefficient. Neurons die at a rate $\frac {\partial N}{\partial t}$, thereby releasing their $A_{\beta }^{i}$. Hence they contribute $A_{\beta }^{i}\left |\frac {\partial N}{\partial t}\right |$ to the growth rate of $A_{\beta }^{o}$, which is the first term on the right-hand side of Eq. (2). The second term on the right-hand side of Eq. (2) represents A *β* constitutively released from APP [5], and the third term accounts for A *β* released by activated astrocytes [16]; *A*_0_ is the reference density of the astrocyte cells in the brain. $A_{\beta }^{0}$ is cleared primarily by peripheral macrophages $\hat {M}_{1}$ and $\hat {M}_{2}$, but also by activated microglias *M*_1_ and *M*_2_, so $d_{A_{\beta }^{o}\hat {M}}>d_{A_{\beta }^{o}{M}}$ [21], and $\hat {M}_{1}$ *M*_1_ are more effective in clearing $A_{\beta }^{o}$ than $\hat {M}_{2}$ and *M*_2_ [46, 47] so 0≤*θ*<1. APP on live neurons shed A *β* peptides both inside the neurons (as $A_{\beta }^{i}$) and outside the neurons (as $A_{\beta }^{o}$). We assume that most $A_{\beta }^{o}$ are produced from dead neurons. Hence, in Eq. (2), we neglected the production of $A_{\beta }^{o}$ by live neurons. We also assumed that ROS increases primarily the A *β* that are within live neurons, and thus neglected the increase of $A_{\beta }^{o}$ by ROS.

#### Equation for ***τ***

Tau protein is constitutively produced at some rate *λ*_*τ*0_. We assume that when $A_{\beta }^{i}$ production exceeds a threshold $A_{\beta }^{i0}$, GSK-3 becomes activated and it mediates hyperphosphorylation of tau. In steady state, the difference $A_{\beta }^{i}-A_{\beta }^{i0}$ is proportional to *R*. Hence the equation for tau is given by:

3 $$\begin{array}{@{}rcl@{}} \frac{\partial \tau}{\partial t}&=&\left(\underbrace{\lambda_{\tau0}+\lambda_{\tau}R}_{production}\underbrace{-d_{\tau} \tau}_{degradation}\right)\frac{N}{N_{0}}.\quad\quad \end{array} $$

We assume that initially we already have a disease state. Thus, in particular, the tau proteins are already hyperphophorylated and ROS induces increases in the production of these proteins.

#### Equations for NFT

The NFTs in neurons (*F*_*i*_) are formed from the hyperphosphorylated tau proteins [4, 6–9], and they are released to the extraceullar space (and are then labeled *F*_0_) when the neurons die [4, 10]. Hence,

4 $$\begin{array}{@{}rcl@{}} \frac{\partial F_{i}}{\partial t}&=&\left(\underbrace{\lambda_{F}\tau}_{production}\underbrace{-d_{F_{i}} F_{i}}_{degradation}\right)\frac{N}{N_{0}},  \end{array} $$

5 $$\begin{array}{@{}rcl@{}} \frac{\partial F_{o}}{\partial t}&=&\underbrace{F_{i}\left|\frac{\partial N}{\partial t}\right|}_{production}\underbrace{-d_{F_{O}} F_{o}}_{degradation}. \end{array} $$

#### Equation for neurons

Hyberphosphorated tau proteins, forming neurofibrillary tangles, cause microtubles depolymerization and destruction, resulting in neuron death [4, 6–9]. Neuron death is also caused by stress from proinflammatory cytokines which is, however, resisted by anti-inflammatory cytokines [20, 22, 23]. For simplicity we represent all the proinflammatory cytokines by TNF- *α* and all the anti-inflammatory cytokines by IL-10. Hence the equation for *N* takes the following form:

6 $$ \begin{aligned} {}\frac{\partial N}{\partial t}=\underbrace{-d_{NF}\frac{F_{i}}{F_{i}+K_{F_{i}}}N-d_{NT}\frac{T_{\alpha}}{T_{\alpha}+K_{T_{\alpha}}}\frac{1}{1+\gamma I_{10}/{K}_{I_{10}}}N}_{death}, \end{aligned}  $$

where the death rates of *N* caused by *F*_*i*_ and *T*_*α*_ are assumed to depend on their saturation levels.

#### Equation for astrocytes

Astrocytes are activated primarily by extracellular TNF- *α* [14, 15], but also by $A_{\beta }^{o}$ [10, 16], so that

7 $$\begin{array}{@{}rcl@{}} \frac{\partial A}{\partial t}&=&\underbrace{\lambda_{AA_{\beta}^{o}} A_{\beta}^{o}+\lambda_{AT_{\alpha}}T_{\alpha}}_{production}\underbrace{-d_{A}A}_{death}. \end{array} $$

#### Equation for dead neurons

The equation for dead neurons, *N*_*d*_, is given by

8 $$ \begin{aligned} &{}\frac{\partial N_{d}}{\partial t}=\underbrace{d_{NF}\frac{F_{i}}{F_{i}+K_{F_{i}}}N+d_{NT}\frac{T_{\alpha}}{T_{\alpha}+K_{T_{\alpha}}}\frac{1}{1+\gamma I_{10}/K_{I_{10}}}N}_{production}\\ &\underbrace{-d_{N_{d}M}\!\left(M_{1}\,+\,M_{2}\right)\frac{N_{d}}{N_{d}\,+\,\bar{K}_{N_{d}}}}_{clearance ~by ~microglia}\underbrace{-d_{N_{d}\hat{M}}\left(\hat{M}_{1}\,+\,\hat{M}_{2}\right)\frac{N_{d}}{N_{d}\,+\,\bar{K}_{N_{d}}}}_{clearance ~by ~macrophages}\!, \end{aligned}  $$

where $\bar {K}_{N_{d}}$ is a Michaelis-Menten coefficient. The first two terms on the right-hand side arise from the death of N cells. The last two terms account for the clearance of *N*_*d*_ by microglias and peripheral macrophages [48].

#### Equation for A ***β***O

The A *β*O are soluble A *β* oligomers and they can diffuse throughout the brain tissue [49, 50]. Their density *A*_*O*_ satisfies the equation:

9 $$\begin{array}{@{}rcl@{}} \frac{\partial A_{O}}{\partial t}-D_{A_{O}}\Delta A_{O}&=&\underbrace{\lambda_{A_{O}}A_{\beta}^{o}}_{production}\underbrace{-d_{A_{O}} A_{O}}_{degradation}, \end{array} $$

where $\lambda _{A_{O}}$ is the rate by which the *A*_*O*_ are formed from the extracellular amyloid *β* peptides, and $D_{A_{O}}\Delta A_{O}$ accounts for the diffusion of *A*_*O*_.

#### Equation for HMGB-1

In general, when cell death occurs through necrosis, dying cells release HMGB-1 [51]. In AD, HMGB-1 is produced by dying neurons [52–54]. Hence,

10 $$\begin{array}{@{}rcl@{}} \frac{\partial H}{\partial t}-D_{H}\Delta H &=&\underbrace{\lambda_{H} N_{d}}_{production}\underbrace{-d_{H}H}_{degradation}. \end{array} $$

#### Equations for activated microglias

Activated microglias have two phenotypes: proinflammatory *M*_1_ and anti-inflammatory *M*_2_. They satisfy the following equations:

11 $$ \begin{aligned} {}\frac{\partial M_{1}}{\partial t}\,+\,\nabla\cdot(M_{1}\nabla H)&=\underbrace{{M_{G}^{0}}\left[\lambda_{MF}\frac{F_{o}}{F_{o}\,+\,K_{F_{o}}}+\lambda_{MA}\frac{A_{O}}{A_{O}\,+\,K_{A_{O}}}\right]\frac{\beta\varepsilon_{1}}{\beta\varepsilon_{1}\,+\,\varepsilon_{2}}}_{production}\\ &\underbrace{-\lambda_{M_{1}T_{\beta}}\frac{T_{\beta}}{T_{\beta}+K_{T_{\beta}}}M_{1}}_{M_{1}\rightarrow M_{2}}\underbrace{-d_{M_{1}}M_{1}}_{death},\\ \end{aligned}  $$

12 $$ \begin{aligned} {}\frac{\partial M_{2}}{\partial t}+\nabla\cdot(M_{2} \nabla H)&=\underbrace{{M_{G}^{0}}\left[\lambda_{MF}\frac{F_{o}}{F_{o}\,+\,K_{F_{o}}}\,+\,\lambda_{MA}\frac{A_{O}}{A_{O}\,+\,K_{A_{O}}}\right]\frac{\varepsilon_{2}}{\beta\varepsilon_{1}\,+\,\varepsilon_{2}}}_{production}\\ &\underbrace{+\lambda_{M_{1}T_{\beta}}\frac{T_{\beta}}{T_{\beta}+K_{T_{\beta}}}M_{1}}_{M_{1}\rightarrow M_{2}}\underbrace{-d_{M_{2}}M_{2}}_{death}, \end{aligned}  $$

where $\varepsilon _{1}=\frac {T_{\alpha }}{T_{\alpha }+K_{T_{\alpha }}}$ and $\varepsilon _{2}=\frac {I_{10}}{I_{10}+K_{I_{10}}}$.

Microglias can travel in the brain [55]. Activated microglias are chemoattracted to dead neurons [10, 13, 15], more precisely, to the cytokines HMGB-1 produced by *N*_*d*_, and this is represented by the second term of the left-hand side of Eqs. (11), (12). Microglias are activated by extracelluar NFTs [10, 13, 15], and by soluble oligomers *A*_*O*_ [11, 12]. They become of *M*_1_ phenotype under proinflammatory signals from TNF- *α*, and of *M*_2_ phenotype under anti-inflammatory signals from IL-10. These facts are expressed by the first term on the right-hand sides of Eqs. (11), (12); $\frac {\beta \varepsilon _{1}}{\beta \varepsilon _{1}+\varepsilon _{2}}$ is the ratio by which the activated microglias become *M*_1_ macrophages, and $\frac {\varepsilon _{2}}{\beta \varepsilon _{1}+\varepsilon _{2}}$ is the ratio by which activated microglias become *M*_2_ macrophages. The parameter *β* reflects the ratio of proinflammatory/anti-inflammatory environment, as determined by the relative ‘strength’ of *T*_*α*_ v.s. *I*_10_.

In addition, there is a transition *M*_1_→*M*_2_ under the TGF- *β* signaling [32], which is accounted by the second term on the right-hand side of these equations.

#### Equations for macrophages

Peripheral macrophages $\hat {M}$ are differentiated from monocytes which migrate through the blood vessels. They satisfy a flux condition

$$\frac{\partial \hat{M}}{\partial n}+\tilde{\alpha}(P)(\hat{M}-M_{0})=0$$

on the boundary of the blood vessels, where *n* is the outward normal, *M*_0_ is the density of the monocytes in the brain capillaries, and $\tilde {\alpha }(P)$ is a function which depends on the concentration of MCP-1. By averaging these fluxes from blood vessels, we can represent (as in [56]) the immigration of $\hat {M}$ macrophages into the brain tissue by a term $\tilde {\alpha }(P)(M_{0}-\hat {M})$. We assume that the incoming macrophages divide into $\hat {M}_{1}$ and $\hat {M}_{2}$ phenotype depending on the relative concentrations of TNF- *α* and IL-10 [47]. Macrophages $\hat {M}_{1}$ can also change phenotype to $\hat {M}_{2}$ macrophages under signaling by TGF- *β*. We finally note that because of the blood-brain barrier (BBB) we do not include diffusion of peripheral macrophages, but we do include chemotaxis by amyloid- *β* plaques or, more specifically, by the soluble *A*_*O*_ [17–19]. Hence peripheral macrophages satisfy the following equations:

13 $$ \begin{aligned} {}\frac{\partial \hat{M}_{1}}{\partial t}\,+\,\nabla\cdot(\hat{M}_{1}\nabla A_{O})&=\underbrace{\alpha(P)(M_{0}\,-\,\hat{M})\frac{\beta\varepsilon_{1}}{\beta\varepsilon_{1}\,+\,\varepsilon_{2}}}_{production}\underbrace{-\lambda_{\hat{M}_{1}T_{\beta}}\frac{T_{\beta}}{T_{\beta}\,+\,K_{T_{\beta}}}\hat{M}_{1}}_{M_{1}\rightarrow M_{2}}\underbrace{-d_{\hat{M}_{1}}\hat{M}_{1}}_{death},\\ \end{aligned}  $$

14 $$ \begin{aligned} {}\frac{\partial \hat{M}_{2}}{\partial t}\,+\,\nabla\cdot(\hat{M}_{2}\nabla A_{O}) &\,=\,\underbrace{\alpha(P)(M_{0}-\hat{M})\frac{\varepsilon_{2}}{\beta\varepsilon_{1}+\varepsilon_{2}}}_{production}\underbrace{\!+\lambda_{\hat{M}_{1}T_{\beta}}\frac{T_{\beta}}{T_{\beta}\,+\,K_{T_{\beta}}}\hat{M}_{1}}_{M_{1}\rightarrow M_{2}}\underbrace{-d_{\hat{M}_{2}}\hat{M}_{2}}_{death},\\ \end{aligned}  $$

where $\hat {M}=\hat {M}_{1}+\hat {M}_{2}$ and $\alpha (P)=\alpha \frac {P}{P+K_{P}}$ [56].

#### Equations for TGF- ***β***, TNF- ***α*** MCP-1 and IL-10

*T*_*β*_ and IL-10 are produced by *M*_2_ microglia and $\hat {M}_{2}$ macrophages. TNF- *α* is produced by proinflammatory macrophages *M*_1_ and $\hat {M}_{1}$. Hence the equations for *T*_*β*_, *T*_*α*_ and *I*_10_ have the following form:

15 $$\begin{array}{@{}rcl@{}} {}\frac{\partial T_{\beta}}{\partial t}-D_{T_{\beta}}\Delta T_{\beta}&=&\underbrace{\lambda_{T_{\beta} M}M_{2}+\lambda_{T_{\beta} \hat{M}}\hat{M}_{2}}_{production}\underbrace{-d_{T_{\beta}}T_{\beta}}_{degradation}, \end{array} $$

16 $$\begin{array}{@{}rcl@{}} {}\frac{\partial I_{10}}{\partial t}-D_{I_{10}}\Delta I_{10}&=&\underbrace{\lambda_{I_{10} M}M_{2}+\lambda_{I_{10}\hat{M}}\hat{M}_{2}}_{production}\underbrace{-d_{I_{10}}I_{10}}_{degradation}, \end{array} $$

17 $$\begin{array}{@{}rcl@{}} {}\frac{\partial T_{\alpha}}{\partial t}-D_{T_{\alpha}}\Delta T_{\alpha}&=&\underbrace{\lambda_{T_{\alpha} M_{1}}M_{1}+\lambda_{T_{\alpha} \hat{M}_{1}}\hat{M}_{1}}_{production}\underbrace{-d_{T_{\alpha}}T_{\alpha}}_{degradation}. \end{array} $$

MCP-1 is produced by activated astrocytes [17–19] and by microglias [12], which are assumed to be of *M*_2_ phenotype. Hence

18 $$\begin{array}{@{}rcl@{}} \frac{\partial P}{\partial t}-D_{P}\Delta P&=&\underbrace{\lambda_{P A}A+\lambda_{PM_{2}}M_{2}}_{production}\underbrace{-d_{P}P}_{degradation}. \end{array} $$

The estimates of parameters in Eqs. (1)–(18) are given in “Appendix".

## Results and discussions

We simulate the model (1)-(18) in a rectangular domain *Ω*={(*x*,*y*),0≤*x*≤1,0≤*y*≤1}. We assume that

19 $$ \begin{aligned} A_{O}, \textit{H}, T_{\beta}, I_{10}, T_{\alpha}~ \text{and}~ P~ \text{satisfy periodic boundary conditions}. \end{aligned}  $$

We take initial values, for each variable X, to be below (or above) the expected steady state for X, if X is expected to grow (or decrease) with the progression of the disease. A specific choice is given below, but the simulations do not change, after a short time, with other choices:

20 $$ \begin{aligned} &{}A_{\beta}^{i}\,=\,10^{-6}~g\!/\!ml,A_{\beta}^{o}\,=\,10^{-8}~g/ml,~\tau\,=\,1.37\!\times\!10^{-10}~g/ml,F^{i}\,=\,3.36\!\times\!10^{-10}~g\!/\!ml,\\ &{}F^{o}\,=\,3.36\!\times\!10^{-11}~g\!/\!ml, N\,=\,0.14~g\!/\!ml,A\,=\,0.14~g\!/\!ml,M_{1}\,=\,M_{2}\,=\,0.02~g\!/\!ml,\\ &\quad\hat{M}_{1}\,=\,\hat{M}_{2}\,=\,N_{d}\,=\,0~g\!/\!ml,H=1.3\!\times\!10^{-11}g\!/\!ml,T_{\beta}\,=\,10^{-6}g\!/\!ml,\\ &\qquad \quad T_{\alpha}\,=\,2\!\times\!10^{-5}g\!/\!ml,I_{10}\,=\,10^{-5}~g\!/\!ml,P\,=\,5\!\times\!10^{-9}g\!/\!ml. \end{aligned}  $$

We also prescribe the value of ROS in Eqs. (1), (3) by

21 $$\begin{array}{@{}rcl@{}} R=R(t)=\left\{ \begin{array}{rl} R_{0}\frac{t}{100}&0\leq t\leq 100\\ R_{0}&t>100 \end{array}\right.. \end{array} $$

**Fig. 2:**
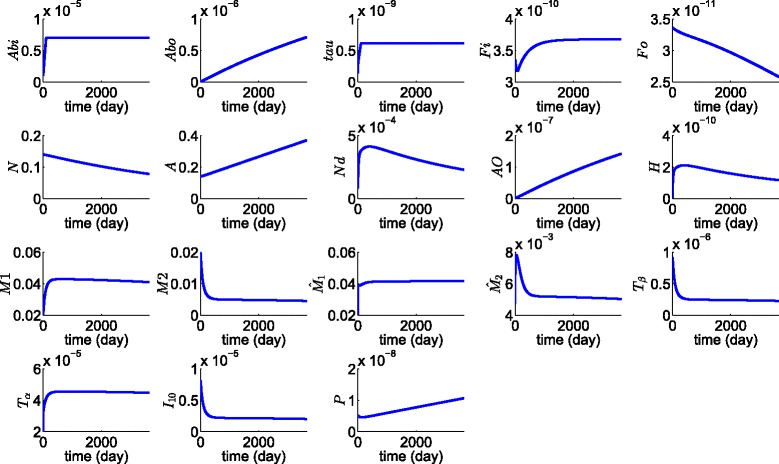
Average concentration of cytokines and average density of cells. All the parameters are as in Tables 2 and 3

Figure 2 shows the average density of all the 18 variables of the model over a period of 10 years. We first observe that, for all the species that tend to a steady state in Fig. 2, the steady states are approximately the same as those that we assumed in estimating some of the model parameters. Thus the steady state values of $\tau,F_{i}, H, M_{1},M_{2},\hat {M}_{1},\hat {M_{2}},T_{\beta },T_{\alpha }$ and *I*_10_ are approximately equal to the values assumed in “Appendix”. We conclude that estimates of the parameters which were based on steady state assumptions on macrophages, microglias and the half-saturation parameters are consistent with the simulation results.

**Table 2:** Parameters’ description and value

Parameter	Description	Value
$D_{A_{O}}$	Diffusion coefficient of A *β*O	4.32×10^−2^ *c* *m* ^2^ day ^−1^ estimated
*D*_*H*_	Diffusion coefficient of HMGB-1	8.11×10^−2^ *c* *m* ^2^ day ^−1^ estimated
$D_{T_{\alpha }}$	Diffusion coefficient for TNF- *α*	6.55×10^−2^ *c* *m* ^2^ day ^−1^ estimated
$d_{T_{\beta }}$	Diffusion coefficient of TGF- *β*	6.55×10^−2^ *c* *m* ^2^ day ^−1^ estimated
$D_{I_{10}}$	Diffusion coefficient of IL-10	6.04×10^−2^ *c* *m* ^2^ day ^−1^ estimated
*D*_*P*_	Diffusion coefficient of MCP-1	1.2×10^−1^ *c* *m* ^2^ day ^−1^ estimated
$\lambda _{\beta }^{i} $	Production rate of $A_{\beta }^{i}$	9.51×10^−6^ g/ml/day estimated
*λ*_*N*_	Production rate of $A_{\beta }^{o}$ by neuron	8×10^−9^ g/ml/day estimated
*λ*_*A*_	Production rate of $A_{\beta }^{o}$ by astrocytes	8×10^−10^ g/ml/day estimated
*λ*_*τ*0_	Production rate of tau proteins in health	8.1×10^−11^ g/ml/day estimated
*λ*_*τ*_	Production rate of tau proteins by ROS	1.35×10^−11^ g/ml estimated
*λ*_*F*_	Production rate of NFT by tau	1.662×10^−3^/day estimated
$\lambda _{AT_{\alpha }}$	Production/activation rate of astrocytes by TNF- *α*	1.54/day estimated
$\lambda _{AA_{\beta }^{o}}$	Production/activation rate of astrocytes by $A_{\beta }^{o}$	1.793/day estimated
$\lambda _{A_{O}}$	Production rate of A *β*O	5×10^−2^/day estimated
*λ*_*H*_	Production rate of HMGB-1	3×10^−5^/day estimated
*λ*_*MF*_	Production/activation rate of microglias by NFT	2×10^−2^/day estimated
*λ*_*MA*_	Production/activation rate of microglias by astrocytes	2.3×10^−3^/day estimated
$\lambda _{M1T_{\beta }}$	Rate of *M*_1_→*M*_2_	6×10^−3^/day estimated
$\lambda _{\hat {M}_{1}T_{\beta }}$	Rate of $\hat {M}_{1}\rightarrow \hat {M}_{2}$	6×10^−4^/day estimated
$\lambda _{T_{\beta } M}$	Production rate of TGF- *β* by M	1.5×10^−2^ day ^−1^ [56, 99]
$\lambda _{T_{\beta }\hat {M}}$	Production rate of TGF- *β* by $\hat {M}$	1.5×10^−2^ day ^−1^ [56, 99]
$\lambda _{T_{\alpha } M1}$	Production rate of TNF- *α* by *M*_1_	3×10^−2^ day ^−1^ estimated
$\lambda _{T_{\alpha } \hat {M}_{1}}$	Production rate of TNF- *α* by $\hat {M}_{1}$	3×10^−2^ day ^−1^ estimated
$\lambda _{I_{10}M_{2}}$	Production rate of IL-10 by *M*_2_	6.67×10^−3^ day ^−1^ [47, 90]
$\lambda _{I_{10}\hat {M}_{2}}$	Production rate of IL-10 by $\hat {M}_{2}$	6.67×10^−3^ day ^−1^ [47, 90]
*λ*_*PA*_	Production rate of MCP-1 by astrocytes	6.6×10^−8^ day ^−1^ estimated
$\lambda _{PM_{2}}$	Production rate of MCP-1 by *M*_2_	1.32×10^−7^ day ^−1^ estimated
*θ*	*M*_2_/*M*_1_ effectivity in clearance of $A_{\beta }^{o}$	0.9 estimated
*α*	Flux rate of macrophages	5 estimated
*β*	Proinflammatory/anti-inflammatory ratio	10 estimated
*γ*	*I*_10_ inhibition ratio	1 estimated

**Table 3:** Parameters’ description and value

Parameter	Description	Value
$d_{A_{\beta }^{i}}$	Degradation rate of $A_{\beta }^{i}$	9.51/day [82]
$d_{A_{\beta }^{o}}$	Degradation rate of $A_{\beta }^{o}$	9.51/day [82]
$d_{A_{\beta }^{o}{M}}$	Clearance rate of $A_{\beta }^{o}$ by microglia	2×10^−3^/day estimated
$d_{A_{\beta }^{o}\hat {M}}$	Clearance rate of $A_{\beta }^{o}$ by macrophages	10^−2^/day estimated
*d*_*τ*_	Degradation rate of tau proteins	0.277/day [88]
$d_{F_{i}}$	Degradation rate of intracellular NFT	2.77×10^−3^/day estimated
$d_{F_{o}}$	Degradation rate of extracellular NFT	2.77×10^−4^/day estimated
*d*_*N*_	Death rate of neurons	1.9×10^−4^/day estimated
*d*_*NF*_	Death rate of neurons by NFTs	3.4×10^−4^/day estimated
*d*_*NT*_	Death rate of neurons by TNF- *α*	1.7×10^−4^/day estimated
$d_{N_{d}M}\phantom {\dot {i}\!}$	Clearance rate of dead neurons by M	0.06/day estimated
$d_{N_{d}\hat {M}}$	Clearance rate of dead neurons by $\hat {M}$	0.02/day estimated
*d*_*A*_	Death rate of astrocytes	1.2×10^−3^ day ^−1^ estimated
$d_{{M}_{1}}\phantom {\dot {i}\!}$	Death rate of *M*_1_ microglias	0.015 day ^−1^ [47, 74]
$d_{{M}_{2}}\phantom {\dot {i}\!}$	Death rate of *M*_2_ microglias	0.015 day ^−1^ [47, 74]
$d_{\hat {M}_{1}}\phantom {\dot {i}\!}$	Death rate of *M*_1_ macrophages	0.015 day ^−1^ [47, 74]
$d_{\hat {M}_{2}}\phantom {\dot {i}\!}$	Death rate of *M*_2_ macrophages	0.015 day ^−1^ [47, 74]
$D_{A_{O}}$	Degradation rate of A *β*O	0.951/day estimated
*d*_*H*_	Degradation rate of HMGB-1	58.71/day [95]
$D_{T_{\alpha }}$	Degradation rate of TNF- *α*	55.45 day ^−1^ [47, 74]
$d_{T_{\beta }}$	Degradation rate of TGF- *β*	3.33×10^2^ day ^−1^ [56, 99]
$d_{I_{10}}\phantom {\dot {i}\!}$	Degradation rate of IL-10	16.64 day ^−1^ [47]
*d*_*P*_	Degradation rate of MCP-1	1.73 day ^−1^[47, 74]
*R*_0_	Initial inflammation by ROS	6 estimated
*M*_0_	Monocytes concentration in blood	5×10^−2^ estimated
*N*_0_	Reference density of neuron	0.14 *g*/*c* *m* ^3^ estimated
${M_{G}^{0}}\phantom {\dot {i}\!}$	Source of microglia	0.047 *g*/*c* *m* ^3^ estimated
*A*_0_	Reference density of astrocytes	0.14 *g*/*c* *m* ^3^ estimated
$\bar {K}_{A_{\beta }^{o}}$	Michaelis-Mention coefficient for $A_{\beta }^{o}$	7×10^−3^ g/ *c* *m* ^3^ estimated
$\bar {K}_{N_{d}}$	Michaelis-Mention coefficient for *N*_*d*_	10^−3^ g/ml estimated
$K_{I_{10}}\phantom {\dot {i}\!}$	Half-saturation of IL-10	2.5×10^−6^ g/ *c* *m* ^3^ estimated
$K_{T_{\beta }}\phantom {\dot {i}\!}$	Half-saturation of TGF- *β*	2.5×10^−7^ g/ml [90]
*K*_*M*_	Half-saturation of microglias	0.047 g/ml estimated
$K_{\hat {M}}$	Half-saturation of macrophages	0.047 g/ml estimated
$K_{M_{1}}\phantom {\dot {i}\!}$	Half-saturation of *M*_1_ microglias	0.03 g/ml estimated
$K_{M_{2}}\phantom {\dot {i}\!}$	Half-saturation of *M*_2_ microglias	0.017 g/ml estimated
$K_{\hat {M}_{1}}$	Half-saturation of $\hat {M}_{1}$ macrophages	0.04 g/ml estimated
$K_{\hat {M}_{2}}$	Half-saturation of $\hat {M}_{2}$ macrophages	0.007 g/ml estimated
$K_{F_{i}}\phantom {\dot {i}\!}$	Half-saturation of intracellular NFTs	3.36×10^−10^ g/ml [89]
$K_{F_{o}}\phantom {\dot {i}\!}$	Average of extracellular NFTs	2.58×10^−11^ g/ml estimated
$K_{A_{O}}\phantom {\dot {i}\!}$	Average of of A *β*O	1×10^−7^ g/ml estimated
*K*_*P*_	Half-saturation of MCP-1	6×10^−9^ g/ml estimated
$K_{T_{\alpha }}$	Half-saturation of TNF- *α*	4×10^−5^ g/ml estimated

We next observe that neurons are dying at approximately the rate of 5% a year, which was one of our important assumptions that was based on clinical data. We also note that, as the disease progresses, the plaque of A *β* peptides, $A_{\beta }^{o}$, and the soluble A *β* oligomers, *A*_*O*_, are increasing; $A_{\beta }^{o}$ reaches the level of 7×10^−6^ g/ml, in agreement with clinical data [57], and the assumed average of *A*_*O*_ concentration, $K_{A_{O}}$, is indeed in good approximation to the average of the profile of *A*_*O*_ in Fig. 2. The assumed average of the *F*_*o*_ concentration, $K_{F_{o}}$, is also in good agreement with the average of the profile of *F*_*o*_ in Fig. 2.

We note that *N*_*d*_ nearly stabilizes over time, at the level assumed in “Appendix," which means that, over time, macrophages and microglias clear debris of dead cells at nearly the same rate at which neurons are dying. Hence $\Big |\frac {\partial N_{d}}{\partial t}\Big |$ becomes very small over time, resulting in significant decline in extracellular NFT, while intracellular NFTs (*F*_*i*_) maintain a comparatively high level.

We finally note that the density of activated astrocytes is slightly increasing in agreement with a mouse model [58] which reports that astrocytes become increasingly prominent with the progression of the disease. The increase in A causes P also to increase, and the average of P is approximately equal to our estimate of *K*_*P*_ in S.I.

### Anti-Alzheimer drugs

Until now, all clinical trials aimed to develop drugs that can cure AD have failed. There are currently no drugs that can prevent, stop or even delay the progression of Alzheimer’s disease, and there are many ongoing clinical trials. According to the 2016 Alzheimer’s Disease Facts and Figures, and the National Institute of Aging, if no cure is found, by 2050 the number of alzheimer’s patients in the U.S. will reach 15 millions and the cost of caring for them will exceed $ 1 trillion annually.

Avenues for AD therapies include prevention of build up of plaque (anti-amyloid drugs), preventing tau aggregation, and reducing inflammation. Clinical trials are concerned with both safety and efficacy. Here we shall use our mathematical model to conduct *in silico* trials with several drugs, addressing only the question of efficacy.

Treatment for AD causes changes in the densities of cells and concentrations of cytokines. In order to determine the efficacy of a drug, we should observe (i) to what extend it decreases the death rate of *N*, since slowing the death of neurons will improve cognition of patients; and (ii) to what extend it decreases $A_{\beta }^{o}$, since A *β* aggregation mediates rapid dysfunction of synaptic plasticity and dendritic channels thereby causing memory loss [36–39].

#### TNF- ***α*** inhibitor

Since TNF- *α* is implicated in generating neurotoxicity which leads to death of neurons, TNF- *α* inhibitor (etanercept) has been considered as a drug for Alzheimer’s patients [59]. In 2015 clinical trials phase 2 [60] the drug has shown some favorable trends but with “no statistically significant changes in cognition.” Since there were no serious adverse events, it was suggested that a larger, broader group needs to be tested before recommending etanercept for use for general Alzheimer patients.

We shall apply our model to determine how this TNF- *α* inhibitor affects AD patients. We use the following procedure:

- Run the model for 300 days in order to ensure that AD has been diagnosed in patients;
- Apply continuous treatment by the drug from day 300 until the end of 10 years.

**Fig. 3:**
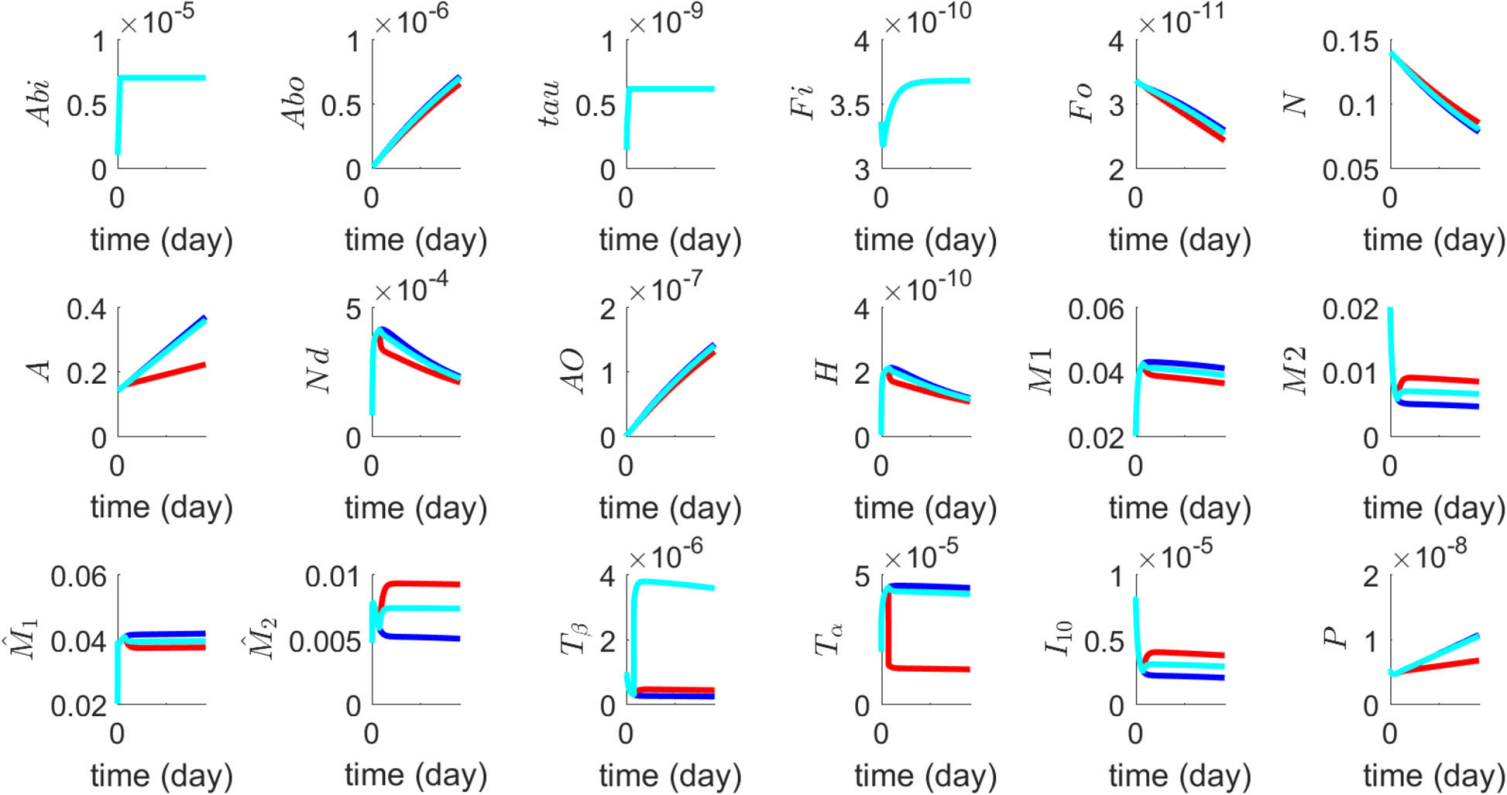
Anti-TNF- *α* drug (*red*), etanercept, with $f=10d_{T_{\alpha }}$; TGF- *β* injection. (*light-blue*) with $g=10d_{T_{\beta }}K_{T_{\beta }}$. *Dark-blue color* corresponds to no treatment, and where several profiles nearly coincide, they are all colored by *light-blue*. All the other parameters are as in Tables 2 and 3

During treatment, the effect of the drug is to replace Eq. (17) for TNF- *α* by the equation

22 $$ \begin{aligned} {}\frac{\partial T_{\alpha}}{\partial t}-D_{T_{\alpha}}\Delta T_{\alpha}\!={\lambda_{T_{\alpha} M_{1}}M_{1}+\lambda_{T_{\alpha} \hat{M}_{1}}\hat{M}_{1}}{-d_{T_{\alpha}}T_{\alpha}}-fT_{\alpha}, \end{aligned}  $$

where *f* is proportional to the amount of etanercept. We note that since etanercept is a soluble TNF receptor fusion protein, it stabilizes TNF- *α* [61] and thus TNF- *α* is diminished at rate *f* *T*_*α*_. The red profiles in Fig. 3 show the result of the treatment with $f=10d_{T_{\alpha }}$, compared to no treatment.

#### TGF- ***β*** injection

TGF- *β* is an anti-inflammatory cytokine which induces phenotype change from proinflammatory to anti-inflammatory macrophages. It was suggested that TGF- *β* mitigates AD pathology [29–34].

We note that the effect of *T*_*β*_ injection is to decrease *M*_1_ and $\hat {M}_{1}$ (see Eqs. (11), (13)), which results in a decrease in *T*_*α*_ (by Eq. (17)) and hence in a decrease in neuronal death rate. To model the treatment by injection of TGF- *β* we replace Eq. (15) for *T*_*β*_ by the equation

23 $$ {}\begin{aligned} \frac{\partial T_{\beta}}{\partial t}-D_{T_{\beta}}\Delta T_{\beta}&=&{\lambda_{T_{\beta} M}M_{2}+\lambda_{T_{\beta} \hat{M}}\hat{M}_{2}}{-d_{T_{\beta}}T_{\beta}}+g, \end{aligned}  $$

where *g* is proportional to the amount of injected TGF- *β*. In steady state, *T*_*β*_ maintains the level of $K_{T_{\beta }}$, while its degradation rate is $d_{T_{\beta }}$. Hence the source of *T*_*β*_ in steady state is $d_{T_{\beta }}T_{\beta }$. We take *g* to be 10 times this source, that is $g=10d_{T_{\beta }}K_{T_{\beta }}$. We then follow the same treatment procedure for TNF- *α* inhibitor. The light-blue profiles in Fig. 3 show the results of the treatment, compared to no treatment.

#### Anti-A ***β*** drugs

There are several drugs in Phase 3 clinical trials that aim to reduce the effect of A *β* aggregation [62]. Among them is aducanumab, which is thought to be microglia-mediated phagocytosis and clearance of A *β* [63, 64]. In our model, this drug will cause a decrease in the concentration of soluble $A_{\beta }^{o}$, by replacing Eq. (2) by the equation

24 $$ \begin{aligned} {}\frac{\partial A_{\beta}^{o}}{\partial t}&={A_{\beta}^{i}\left|\frac{\partial N}{\partial t}\right|+\lambda_{N}\frac{N}{N_{0}}+\lambda_{A}\frac{A}{A_{0}}}\\ &{-\left(d_{A^{o}_{\beta} \hat{M}} (\hat{M}_{1}\!+\theta\hat{M}_{2})+d_{A^{o}_{\beta} {M}}({M}_{1}+\theta{M}_{2})(1\,+\,h)\right)\frac{A_{\beta}^{o}}{A_{\beta}^{o}+\bar{K}_{A_{\beta}^{o}}}}, \end{aligned}  $$

where *h* is proportional to the amount of the dozing level; we take *h*=10.

**Fig. 4:**
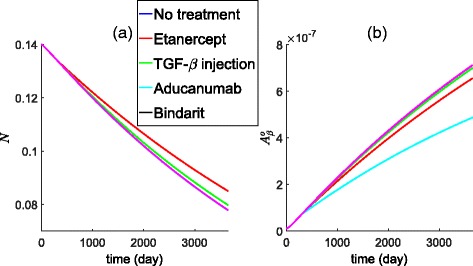
Treatment with etanercept (decreasing *T*_*α*_ degradation rate by 10 fold), TGF- *β* injection (increasing its constitutive source by 10 fold), aducanumab (increasing the clearance rate of $A_{\beta }^{o}$ by 10 fold), and bindarit (increasing MCP-1 natural degradation by 10 fold). In **a**, the profiles of no treatment, bindarit and aducanumab coincide. In **b**, no treatment and bindarit coincide. The lowest curve in Fig. 4 a, and the highest curve in Fig. 4 b, correspond to the case where the curves of no treatment and several other drugs coincide; these drugs have negligible efficacy

Figure 4 shows the efficacy of several drugs in terms of *N* and $A_{\beta }^{o}$. The lowest curve in Fig. 4 a, and the highest curve in Fig. 4 b, correspond to the case where the curves of no treatment and several other drugs coincide; these drugs have negligible efficacy. Following the same treatment procedure as in the case of TNF- *α* inhibitor, Fig. 4 shows no efficacy of aducanumab in terms of *N* but significant efficacy in terms of $A_{\beta }^{o}$ in comparison to no treatment and to treatments by TNF- *α* inhibitor and TGF- *β* injection.

#### MCP-1 inhibitor

Bindarit was shown to inhibit CCL2 (MCP-1) in brain tissue [65]. Hence it decreases $\hat {M}_{1}$ (by Eq. (13)), which results in a decrease in *T*_*α*_ (by Eq. (17)) and thus also in a decrease in neuronal death rate. Bindarit was also reported to inhibit A *β*-induced neuronal death in vitro [66]. Hence it has a therapeutic potential in the treatment of neuroninflammatory/neurodegenerative diseases like AD [66]. We can conduct *in silico* trial with bindarit by revising Eq. (18) for *P*, replacing it with the equation

25 $$\begin{array}{@{}rcl@{}}\frac{\partial P}{\partial t}-D_{P}\Delta P&=&{\lambda_{P A}A+\lambda_{PM_{2}}M_{2}}{-d_{P}P}(1+k) \end{array} $$

with *k*=10 Following the treatment procedure as in the case of of TNF- *α* inhibitor, Fig. 4 shows no efficacy of the drug in terms of *N* and $A_{\beta }^{o}$ in comparison to no treatment.

Methylthiomnium chloride (MTC) is the first identified tau aggregation inhibitor currently in Phase 3 trial [27]. In our model the drug will cause a decrease in the production of tau proteins and in their ability to turn into NFT. We model this by multiplying the production terms *λ*_*τ*0_ and *λ*_*τ*_ by 1/10. Following the procedure as in case of TNF- *α* inhibitor, we found that the drug has almost negligible efficacy (not shown here).

#### Combination therapy

**Fig. 5:**
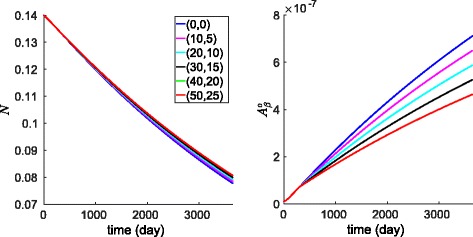
Combined treatment with etanercept fold number f and aducanumab fold number h for several values of (*f, h*)

The results of Fig. 4 suggest that a combination therapy with etanercept (TNF- *α* inhibitor) and aducanumab (anti-A *β* drug), under the same ’10-fold’ amount, could both slow the death rate of neurons and decrease the growth of A *β* in a significant way. Figure 5 shows the dynamics of *N* and $A_{\beta }^{o}$ under such combination of drugs with different proportions of fold numbers: (etanercept,aducanumab)=(0,0) (no drugs), (10,5), (20,10), (30,15), (40,20) and (50,25). The reduction in the death of neurons, after 10 years, compared to the case of no drugs, is 3.8, 5.2, 6.4, 7.9 and 9.2%, respectively, and the respective reduction in the concentration of A *β* is 21, 32.2, 43.6, 53.9 and 64.1%.

We next consider combined therapy for any value of etanercept (f) and aducanumab (h). We define the *N*-efficacy of (f, h), *E*_*N*_(*f*,*h*), to be

$$E_{N}(f,h)=\frac{N(f,h)-N(0,0)}{N(0,0)}, $$

where the density *N* is computed at the end of 10 years. Similarly we define the anti-$A_{\beta }^{o}$ efficacy by

$$E_{A_{\beta}}(f,h)=\frac{A_{\beta}^{o}(0,0)-A_{\beta}^{o}(f,h)}{A_{\beta}^{o}(0,0)} $$

where the $A_{\beta }^{o}$ concentration is computed also at the end of 10 years.

**Fig. 6:**
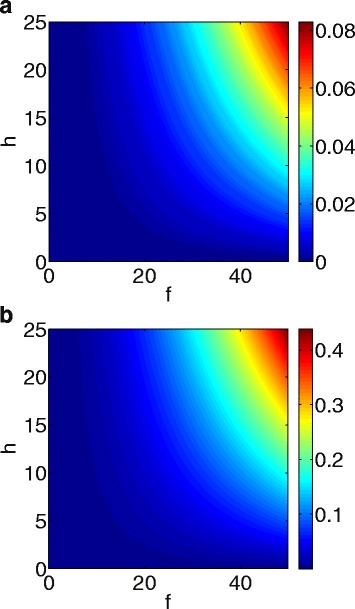
Efficacy maps. Etanercept (with fold number *f*) varies along the horizontal axis, and aducanumab (with fold number *h*) varies along the vertical axis. The column vector indicates the efficacy of treatment for any pair (*f,h*): **a** N-efficacy; **b** Anti-$A_{\beta }^{o}$ efficacy

Figure 6 is an efficacy map of the combined therapy with *f* in range of (0,50) and h in the range of (0,25). For any pair (f,h) the color columns in Fig. 6a and 6b show the efficacy for N and anti-$A_{\beta }^{o}$.

We see that the efficacy of the combined therapy is very small if *f*<20 or *h*<10, and it increases sharply with f and h in the region where {40<*f*<50,20<*h*<25}.

From Fig. 6 we see that anti-A *β* antibody decreases the external concentration of A *β* ($A_{\beta }^{o}$) with efficacy less than 0.5 (h=20, f=0). Higher efficacy requires *T*_*α*_ inhibitor (h=20, f=20) which will protect neuron from death and prevent astrocytes activation, and thereby reduce $A_{\beta }^{o}$. This result can be explained by our assumptions in Eq. (2) where we neglected the production of $A_{\beta }^{o}$ by live neurons and the increase of $A_{\beta }^{o}$ by ROS.

The PK/PD literature employs the concept of combination index (*ϕ*) in order to assess the level of synergy between two drugs [67]. This concept was used in simulations of several diseases (e.g. cancer and microbial diseases) in order to determine optimal dosage regimens [67–69]. Since in our AD model it is not clear how to define *ϕ*, and no data are available to evaluate *ϕ*, we shall, instead, introduce the following concept, for example in the case of etanercept and aducanumab:

We say that these two drugs at concentrations f and g have positive synergy with respect to N if *E*_*N*_(*f*,*g*)>*E*_*N*_(2*f*,0) and *E*_*N*_(*f*,*g*)>*E*_*N*_(0,2*g*). We accordingly define the synergy index *σ*_*N*_=*σ*_*N*_(*f*,*g*) by

$$\sigma_{N}=E_{N}(f,g)/\max\{E_{N}(2f,0),E_{N}(0,2g)\}. $$

**Fig. 7:**
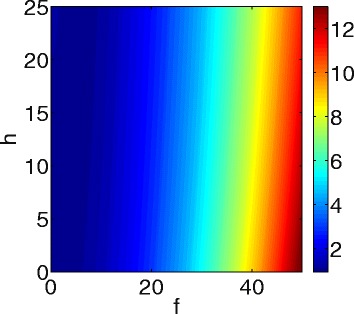
Synergy map for combination therapy with etanercept (*f*) and aducanumab (*h*)

Thus, *σ*_*N*_>1 means positive synergy and *σ*_*N*_<1 means negative synergy. The above definition depends on the doses f, g. If *σ*_*N*_ is large then the combination therapy at total amount *f*+*g* is much more effective than a single therapy, at the total same amount, in reducing the death rate of N. If *σ*_*N*_<1 then a single drug is preferable. Similarly one can define the synergy index $\sigma _{A_{\beta }^{o}}$ with respect to $A_{\beta }^{o}$. Figure 7 shows the synergy index *σ*_*N*_ for (f, g) in the range 0<*f*<50 and 0<*g*<25. We see that there is a positive synergy between etanercept (f) and aducanumab (h). Furthermore, given a total amount A of the combined drugs, so that f+h=A, the synergy increases as f/g increases. This suggests that in an optimal regimen f should be significantly larger than h, provided negative side-effects are discounted.

The synergy map for $\sigma _{A_{\beta }^{o}}$ is similar to that of *σ*_*N*_ (not shown here), and so the synergy increases when f/g is increased.

From Fig. 5 we see that although the amyloid level are controlled, cell death levels do not decrease significantly. This may suggest that other combinations of drugs may target complimentary pathways more efficiently. For example, it was suggested in [70] that Amyloid *β* and tau combine to induce neuron into cell cycle, which leads to cell death; accordingly, one could explore using anti-A *β* and anti tau aggregation in combination therapy.

## Sensitivity analysis

Sensitivity analysis on the model parameters can support the robustness of the simulation results. But it can also suggest what drugs do not work and what drugs are more likely to work. We conducted sensitivity analysis on parameters associated with production and removal rates of $A_{\beta }^{o}$, death rates of N, and production rates of TNF- *α*, TGF- *β* and MCP-1:

$$\begin{aligned} {}&\lambda_{N},\lambda_{A},d_{NF},d_{NT},(\lambda_{T_{\alpha} M_{1}},\lambda_{T_{\alpha}\hat{M}_{1}})\gamma,(\lambda_{T_{\beta} M},\lambda_{T_{\beta}\hat{M}}) \\&\quad\times\delta,(d_{A_{\beta}^{o} M},(\lambda_{PA},\lambda_{PM_{2}})\xi,d_{A_{\beta}^{o} \hat{M}})\epsilon, \end{aligned} $$

where we varied *λ*_*N*_, *λ*_*A*_, *d*_*NF*_, *d*_*NT*_ between $\frac {1}{2}$ and twice their value in Tables 2 and 3, and varied *γ*, *δ*, *ξ*, *ε* between $\frac {1}{2}$ and 2.

**Fig. 8:**
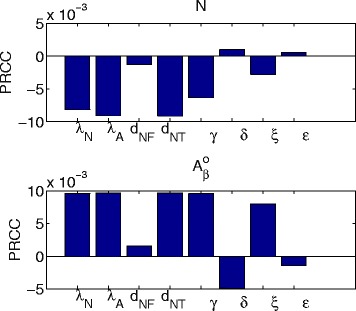
The PRCC values of parameter for sensitivity analysis

Following the sensitivity analysis method described in [71], we performed Latin hypercube sampling and generated 2000 samples to calculate the partial rank correlation coefficients (PRCC) and p-values with respect to the density of N and with respect to the concentration of $A_{\beta }^{o}$ at time t=10 years. The results are shown in Fig. 8. All the *p*-values were less than 0.01. A positive PRCC (i.e. positive correlation) for N means that an increase in the parameter will increase the number of live neurons. A negative PRCC for N means that an increase in the parameter will decrease the number of live neurons. Similarly, a positive (negative) PRCC for $A_{\beta }^{o}$ means that an increase in the parameter will increase (decrease) the concentration of $A_{\beta }^{o}$. Thus, for example, we see that *d*_*NF*_ and *d*_*NT*_ are negatively correlated to N and positively correlated to $A_{\beta }^{o}$. This is not surprising since, with an increase in *d*_*NF*_ and *d*_*NT*_, more neurons die (so N decreases) and as a result more A *β* emerge from the increasingly dying neurons, thus raising the concentration of $A_{\beta }^{o}$. The fact that the correlation coefficients of *d*_*NT*_ are significantly larger than the correlation coefficients of *d*_*NF*_, suggests that a drug which blocks TNF- *α* would be more effective than a drug which clears the *F*_*i*_. The other PRCC values can also be seen to be consistent with the model dynamics.

We observe that *ε* is negatively correlated to $A_{\beta }^{o}$. Indeed, if *ε* is increased, more $A_{\beta }^{o}$ are cleared out (by Eq. (2)). To see how this affects N we note that if $A_{\beta }^{o}$ is decreased then *A*_*O*_ decreases (by Eq. (9)) and correspondingly *M*_1_ decreases (by Eq. (11)), and then *T*_*α*_ decreases (by Eq. (17)); so we may expect N to increase, but perhaps not much, since we have ignored other indirect interactions from the model. From Fig. 8 we see that *ε* is indeed positively correlated to N but the correlation is small. The correlation levels of *ε* with respect to N and $A_{\beta }^{o}$ suggest that an anti-A *β* drug, like aducanumab, will have some benefits in reducing Amyloid *β*, but little benefit in reducing death of neurons. This is also seen from Fig. 4.

## Conclusion

AD is an irreversible progressive neuroninflammatory/neurodegenerative disease that destroys memory and cognitive skills. Currently there is no drug that can cure, stop, or even slow the progression of the disease. Life expectancy at diagnosis is 10 years, and, at death, 50% of the brain neurons have already died. AD patients show abnormal aggregation of beta-amyloids ($A_{\beta }^{o}$) and neurofibrillary tangles (NFTs) of hyperphosphorylated tau proteins. NFTs destroy microtubles in neurons, which results in neurons death. Soluble $A_{\beta }^{o}$ oligomers activate microglias (the resident macrophages in the brain), thereby initiating inflammatory response. Additionally, peripheral macrophages, responding to cue from MCP-1 produced by astrocytes, are attracted to the brain and increase the inflammatory environment, which is harmful to neurons.

Figure 1 is a schematic network of AD: it includes neurons, astrocytes, microglias, peripheral macrophages, *β*-amyloids, tau proteins, and several cytokines involved in the cross-talk among the cells. In the present paper, we developed a mathematical model of AD based on Fig. 1. The model can be used to explore the efficacy of drugs that may slow the progress of the disease. We conducted several in silico trials with several drugs: etanercept (TNF- *α* inhibitor), injection of TGF- *β*, aducanumab (Anti-A *β* drug) and bindarit (MCP-1 inhibitor). We found that at ’10-fold’ level, etanercept has the largest efficacy in slowing death of neurons, while aducanumab has the largest efficacy in reducing the aggregation of $A_{\beta }^{o}$, although these efficacies were quite small. Based on these findings we propose that clinical trials should use a combination therapy with etanercept (f) and aducanumab (h). In Fig. 6 we developed efficacy maps for any combination therapy with 0<*f*<50 and 0<*h*<25, and we used this map to derive, in Fig. 7, a synergy map for *σ*_*N*_=*σ*_*N*_(*f*,*g*). Figure 7 shows that the synergy between f and g increases if f/g increases, while f+g is kept fixed. This suggests that in an optimal regimen with fixed total amount, A, of the drugs, f should be significantly larger than h. We did not consider here, however, adverse side effects that are likely to limit the amount of drugs that can be given to a patient. When these limits become better known, one could then proceed to determine the optimal combination of etanercept and aducanumab for slowing the progression of AD.

The mathematical model developed in this paper depends on some assumptions regarding the mechanism of interactions involving amyloid, tau and neunofilaments in AD. There are currently not enough data to sort out competing assumptions. Hence the conclusion of the paper regarding combination therapy should be taken with caution.

Our mathematical model focused on the progression of AD in terms of neurons death and amyloid *β* aggregation. But dendritic pathologies also play an important role in the disease. Dendritic abnormalities in AD include dystrophic neuritides, reduction in dendritic complexity and loss in dendritic spines [36, 37]. In particular, A *β* plaques affect dendritic channels, and NFT mediates synaptic dysfunction [36–39]. Recent studies also begin to address white matter degeneracy that could help identify high risk of AD [72].

## Appendix

### Parameter estimation

In the sequel, in an expression of the form $\frac {X}{X+K_{X}}$ in the context of activation, the half-saturation parameter *K*_*X*_ is taken to be the steady state of the species *X* provided *X* tends to a steady state. Hence in a steady state equation this factor is equal to $\frac {1}{2}$. If *X* does not tend to a steady state then the parameter *K*_*X*_ will be taken to be the estimated average of *X* over a period of 10 years, the average survival time of AD patients [73]. In an expression of the form $\frac {1}{1+\gamma X/K_{X}}$ (where *γ*=*γ*(*X*)) in the context of inhibition, *K*_*X*_ is again the half-saturation of *X*, so that in steady state the inhibition is 1/(1+*γ*). If cells *Y* phagocytose species *X*, then the clearing rate is proportional to $Y\frac {X}{X+\bar {K}_{X}}$ where the Michaelis-Menten constant $\bar {K}_{X}$ depends only on the ‘eating capacity’ of *Y*, so $\bar {K}_{X}$ has no relation to the half-saturation of *X*.

#### Diffusion coefficients

The diffusion coefficient of proteins (*Y*) are proportional to $1/M_{Y}^{1/3}$, where *M*_*Y*_ is the molecular weight [74]. Accordingly, we have the following relation [75]:

$$D_{Y}=\frac{M_{V}^{1/3}}{M_{Y}^{1/3}}D_{V}, $$

where *M*_*V*_ and *D*_*V*_ are the molecular weight and the diffusion coefficient of VEGF. Since *D*_*V*_=8.64×10^−2^ *c* *m* ^2^ day ^−1^[76], *M*_*V*_=24 kDa [76], *M*_*P*_=8.9 kDa [77], $M_{T_{\beta }}=55$ kDa [78], $M_{T_{\alpha }}=55$ kDa [79], $M_{I_{10}}=70$ kDa [46], and *M*_*H*_=29 kDa [80], we get *D*_*P*_=1.20×10^−1^ *c* *m* ^2^ *day* ^−1^, $D_{T_{\beta }}=6.55\times 10^{-2}$ *c* *m* ^2^ *day* ^−1^, $D_{T_{\alpha }}=6.55\times 10^{-2}$ *c* *m* ^2^ *day* ^−1^, $D_{I_{10}}=6.04\times 10^{-2}$ *c* *m* ^2^ *day* ^−1^ and *D*_*H*_=8.11×10^−2^ *c* *m* ^2^ *day* ^−1^.

Molecular weight of *A* *β* is 24 kDa [81], so in soluble state its diffusion coefficient would be 8.64×10^−2^ *c* *m* ^2^ *day* ^−1^. We assume that soluble oligomer *A* *β* *O* has a smaller diffusion coefficient, namely, $D_{A_{O}}=4.32\times 10^{-2}$ *c* *m* ^2^ *day* ^−1^.

#### Eq. (1)

By [82], the half-life of $A_{\beta }^{i}$ is 1.5–2 h in mice. Hence $d_{A^{i}_{\beta }}=d_{A^{o}_{\beta }}=\frac {ln 2}{1.75}\times 24$=9.51 /day. Membrane proteins APP shed amyloid *β*, some end up inside the cell and some outside the cell. We assume that in healthy steady state $A^{i}_{\beta }=A^{o}_{\beta }$, however the simulation results do not change appreciably if we take $A^{o}_{\beta }>A^{i}_{\beta }$. According to [57], the density in brain-gray matter of $A_{\beta }^{o}$ is approximately 1000 ng/g in control and 7000 ng/g in AD. Hence, from the steady state of Eq. (1) in a healthy normal case, $A^{i}_{\beta }=10^{-6}$ g/ml and $\lambda _{\beta }^{i}=d_{A_{\beta }^{i}}\times 10^{-6}$= 9.51×10^−6^ g/ml/day. From the steady state of Eq. (1) in AD and Eq. (21) we then get that *R*_0_=6.

The brain has 75% water and 60% of its dry matter is fat. We assume that the average density of brain tissue is 1 *g*/*c* *m* ^3^. The human brain has 100 billion neurons, and its weight is approximately 1400 g, so its volume is approximately 1400 ml. Hence its neurons number density is 7×10^7^ neurons/ *c* *m* ^3^. The diameter of neurons is 16 *μ* *m* [83]. Accordingly, we estimate the volume of 1 neuron to be 2×10^−9^ *c* *m* ^3^, and the neurons density is then 7×10^7^×2×10^−9^ *g*/*c* *m* ^3^, that is *N*_0_=0.14 *g*/*c* *m* ^3^.

#### Eq. (2)

The number of neurons is three times the number of microglia [55], hence $K_{\hat {M}}=\frac {1}{3}N_{0}=0.047$ g/ml.

By [16] an astrocyte produces much less A *β* than a neuron, so we take $\lambda _{A}=\frac {1}{10}\lambda _{N}$.

Microglias are the first responders to NFTs and A *β*O. Peripheral macrophages arrive later, and their immune response may perhaps exceed that of microglia, but this is currently not known [12, 84]. We assume that in steady state the microglias density *M* and the peripheral macrophages density $\hat {M}$ are equal, so that $\hat {M}=K_{\hat {M}}=M=K_{M}=0.047$ g/ml. Motivated by the inflammatory immune attack in AD [85], we assume that, in steady state, the proinflammatory macrophages exceed the anti-inflammatory macrophages, and that proinflammatory peripheral macrophages exceed the proinflammatory microglias. Thus, in steady state, $\hat {M}_{1}>\hat {M}_{2}$, *M*_1_>*M*_2_ and $\hat {M}_{1}>M_{1}$, and we take $K_{\hat {M}_{1}}=0.04$, $K_{\hat {M}_{2}}=0.007$, $K_{M_{1}}=0.03$, $K_{M_{2}}=0.017$.

Activated microglias are poorly phagocytic for A *β* compared to peripheral macrophages [6]. Accordingly we take

$$d_{A_{\beta}^{o}{M}}=\frac{1}{5}d_{A_{\beta}^{o}\hat{M}}. $$

Taking $d_{A_{\beta }^{o}\hat {M}}=10^{-2}$/day, we then have

$$d_{A_{\beta}^{o}{M}}=2\times10^{-3}/day. $$

We assume that $\hat {M}_{1}$ and *M*_1_ are more effective than $\hat {M}_{2}$ and *M*_2_ in clearing A *β*, and take *θ*=0.9.

We assume that survival time of patients with AD is 10 years, and that at the end-stage 50% of their neurons have died [73]. Hence, the death rate of N is $d_{N}=\frac {ln2}{10 ~years}=1.9\times 10^{-4}$/day.

By [57], $A_{\beta }^{o}=7\times 10^{-6}$ g/ml. We assume that the clearance of $A_{\beta }^{o}$ by macrophages and microglias is nearly unlimited (i.e., it is almost linear in $A_{\beta }^{o}$) by taking $\bar {K}_{A_{\beta }^{o}}=10^{3}A_{\beta }^{o}=7\times 10^{-3}$ g/ml. To estimate *λ*_*N*_, we first consider the steady state of Eq. (2),

$$ \begin{aligned} &{}10^{-6}\left|\frac{\partial N}{\partial t}\right|_{\text{average}}+\lambda_{N}+\frac{1}{10}\lambda_{N}=\\ &{}\left(d_{A^{o}_{\beta} \hat{M}} (K_{\hat{M}_{1}}\,+\,0.9K_{\hat{M}_{2}})\,+\,d_{A^{o}_{\beta} {M}}(K_{{M}_{1}}\,+\,0.9K_{{M}_{2}})\right)\frac{A_{\beta}^{o}}{A_{\beta}^{o}\,+\,\bar{K}_{A_{\beta}^{o}}}. \end{aligned}  $$

To estimate the average of $\left |\frac {\partial N}{\partial t}\right |$, we use the equation

$$N(t)=N_{0}e^{-d_{N}t},~N(0)=0.14~g/ml, $$

so that

$$\left|\frac{dN}{dt}\right|=0.14\times1.9\times10^{-4}e^{-1.9\times10^{-4}t}. $$

The values of $\left |\frac {\partial N}{\partial t}\right |$ for 500<*t*<1000 days vary very little, i.e., from 1.8×10^−5^ g/ml/day to 1.9×10^−5^ g/ml/day. We take $\left |\frac {dN}{dt}\right |=1.8\times 10^{-5}$ g/ml/day as the average of $\left |\frac {dN}{dt}\right |$ over 10 years, but other choices do not affect significantly our simulation results. We then get that *λ*_*N*_=4×10^−9^ g/ml/day.

The estimate of *λ*_*N*_ was based on the steady-state assumption in Eq. (2). However, in AD the A *β* peptides are continuously aggregating, so that the steady state assumption needs to be revised. We do this by increasing the value of *λ*_*N*_: we take *λ*_*N*_=2×4×10^−9^= 8×10^−9^ g/ml/day, and then *λ*_*A*_=8×10^−10^ g/ml/day.

The number of astrocytes is approximately equal to the number of neurons [86, 87], hence *A*_0_=*N*_0_=0.14 g/ml.

#### Eq. (3)

Half-life of tau proteins is 60 hours [88]. Hence $d_{\tau }=\frac {ln2}{60/24}=24ln2$=0.277/day. Concentration of tau proteins is in healthy normal individuals is 137 pg/ml and, in AD, 490 pg/ml [89]. From the steady state of Eq. (3) in the healthy case, we have *λ*_*τ*0_=*d*_*τ*_ *τ*, where *τ*=137 pg/ml. Hence *λ*_*τ*0_= 3.78×10^−11^ g/ml/day. Similarly, *λ*_*τ*0_+*λ*_*τ*_ *R*=*d*_*τ*_ *τ* in AD, where *τ*=490 pg/ml. Hence we have *λ*_*τ*_ *R*=8.1×10^−11^ g/ml, or *λ*_*τ*_=1.35×10^−11^/day.

#### Eqs. (4) and (5)

We assume that neurofibrillary tangles inside neurons are much more stable than tau proteins, taking $d_{F_{i}}=\frac {1}{10^{2}}d_{\tau }=2.77\times 10^{-3}$/day. We also assume that extracellular NFTs do not degrade as fast as internalized NFTs, taking $d_{F_{o}}=\frac {1}{10}d_{F_{i}}=2.77\times 10^{-4}$/day.

We also assume that 60% of the hyperphosphorglated tau proteins become neurofibrillary tangles. From the steady state of Eq. (4) we then have that $\lambda _{F}=0.6d_{F_{o}}\phantom {\dot {i}\!}$. Hence *λ*_*F*_=1.662×10^−3^/day.

#### Eq. (6)

It is not known whether the rate of death of neurons caused by NFT is larger or smaller than the death rate caused by *T*_*α*_. We take *d*_*NF*_=2*d*_*NT*_, but the simulation of the model in the case where *d*_*NT*_=2*d*_*NF*_ are very similar (not shown here). Assuming that at steady state of Eq. (6) the concentrations of *F*_*i*_, *T*_*α*_ and *I*_10_ are at half-saturation, we get $d_{NF}\left (\frac {1}{2}+\frac {1}{4}\frac {1}{1+\gamma }\right)=d_{N}$, so that $d_{NF}=\frac {4+4\gamma }{3+2\gamma }\times 1.9\times 10^{-4}$/day and $d_{NT}=\frac {2+2\gamma }{3+2\gamma }\times 1.9\times 10^{-4}$/day. In particular, if *γ*=1 then *d*_*NF*_=2.4×10^−4^/day and *d*_*NT*_=1.7×10^−4^/day. We take $K_{I_{10}}=2\times 10^{-6}$ g/ *c* *m* ^3^ (which is somewhat larger than the estimated half-saturation of *I*_10_ in lung inflammation [47, 90]). We assume that in AD, 60% of hyperphosphorylated tau proteins (whose concentration in disease is 490 pg/ml [89]) are in NFT form, so that $K_{F_{i}}=0.6\times 490$ pg/ml= 2.94×10^−10^ g/ml. In [89] the concentration of tau protein was taken uniformly in the tissue of patients. We assume, however, that the concentration of NFT is higher inside neurons than outside neurons, and take $K_{F_{i}}=3.36\times 10^{-10}$ g/ml, $K_{F_{o}}=2.58\times 10^{-11}$ g/ml. From the steady state of Eq. (17) and the estimates of $\lambda _{T_{\alpha } M_{1}}$ and $\lambda _{T_{\alpha }\hat {M}_{1}}$ (see under Eq. (17) below) we get *T*_*α*_=4×10^−5^ g/ml, so that $K_{T_{\alpha }}=4\times 10^{-5}$ g/ml.

#### Eq. (7)

We take the half-life of astrocytes to be the same as the half-life of ganglionic glial cells, that is, 600 days [91]. Hence *d*_*A*_=1.2×10^−3^/day. We assume that the activation of astrocytes is due more to TNF- *α* than to A *β*, and take $\lambda _{A T_{\alpha }}T_{\alpha }=2\lambda _{AA_{\beta }^{o}}A_{\beta }^{o}$. By the steady state of Eq. (7) we then get $\lambda _{AT_{\alpha }}=1.4$/day, and $\lambda _{AA_{\beta }^{o}}=1.63$/day. Actually, in a mouse model of AD, the number of activated astrocytes is increasing [58]. So we compensate for this by increasing both $\lambda _{AT_{\alpha }}$ and $\lambda _{AA_{\beta }^{o}}$ by a factor 1.1, taking $\lambda _{AT_{\alpha }}=1.54$/day and $\lambda _{AA_{\beta }^{o}}=1.793$/day.

#### Eq. (8)

In mice experiments [92], macrophages phagocytosed apoptotic cells at rates that varied in the range 0.1–1.27/h. We assume that necrotic cells (and their debris) in human brain are phagocytosed by peripheral macrophages at rate $d_{N_{d}\hat {M}}=0.2$/day. We also assume that microglia play a greater role in clearing necrotic neurons, and take $d_{N_{d}M}=3\times 0.2$=0.6/day. We also take $\bar {K}_{N_{d}}=10^{-3}$ g/ml.

#### Eq. (9)

We assume the degradation rate of *A*_*O*_ is much slower than that of $A_{\beta }^{o}$, taking $d_{A_{O}}=\frac {1}{10}d_{A_{\beta }^{o}}=0.951$/day. The ratio of soluble *A*_*O*_ to total $A_{\beta }^{o}$ is approximately $\frac {1}{25}$ [93].

From the steady state of Eq. (9) we then get $\lambda _{A_{O}}=\frac {1}{25}d_{A_{O}}=3.8\times 10^{-2}$/day.

The estimate of $\lambda _{A_{O}}$ was based on the steady-state assumption in Eq. (9). However, in AD the soluble A *β* oligomer is continuously increasing, following the increase in $A_{\beta }^{o}$, so the steady-state assumption needs to be revised. We do this by increasing the above value of *λ*_*AO*_, taking the new value to be *λ*_*AO*_=5×10^−2^/day.

#### Eq. (10)

Concentration of HMGB-1 in neurons is 1.3 *n* *g*/*m* *l* [94], hence *H*=0.14×1.3 ng/ml= 1.8×10^−10^ g/ml. Half-life of HMGB-1 is 17 minutes [95], so that *d*_*H*_=58.71/day. We assume that *N*_*d*_ stabilizes somewhere below 2.5×10^−4^ g/ml. From the steady state of Eq. (10), we then get *λ*_*H*_=3×10^−5^/day.

#### Eqs. (11) and (12)

We take $d_{M_{1}}=d_{M_{2}}=0.015$/day [47, 90]. Then, our assumption (under Eq. (2)) that $K_{M_{1}}>K_{M_{2}}$ suggests that *β*>1. We take *β*=10.

We take ${M_{G}^{0}}=K_{M}=0.047$ g/ml and *α*=5. In the absence of data, we take the production rate *λ*_*MF*_ of macrophages by NFT to be the same as the production rate under stimulation by *M. Tuberculosis* in [90], namely, *λ*_*MF*_=2×10^−2^/day. We assume that production rate of macrophages by NFT is larger than the production rate by *A*_*O*_, and take *λ*_*MA*_=2.3×10^−3^/day.

By [57] the concentration of A *β* in AD is 7×10^−6^ g/ml and, by [55], the ratio of *A*_*O*_ to $A_{\beta }^{o}$ is $\frac {1}{25}$, so that $K_{A_{O}}=\frac {1}{25}\times 7\times 10^{-6}=2.8\times 10^{-7}$ g/ml.

We assume that more NFT reside within neurons than outside them, so that $K_{F_{o}}$ is smaller than $K_{F_{i}}$. Recalling that $K_{F_{i}}=3.36\times 10^{-10}$ g/ml, we take $K_{F_{o}}=2.58\times 10^{-11}$ g/ml.

The coefficient $\lambda _{{M}_{1}T_{\beta }}\phantom {\dot {i}\!}$ is the rate by which TGF- *β* affects the change of phenotype from *M*_1_ to *M*_2_. In the case of infection in the lung by *M. tuberculosis*, under inflammatory conditions caused by the pathogen, $\lambda _{M_{1}T_{\beta }}=6\times 10^{-3}$/day [90]; we take it to be the same in the present case. We take $K_{T_{\beta }}=2.5\times 10^{-7}$ g/ml, and $K_{I_{10}}=2.5\times 10^{-6}$ g/ml.

#### Eqs. (13) and (14)

Peripheral macrophages immigrate into the brain of AD [96, 97]. We assume that, because of the BBB, the concentration of monocytes in the brain capillaries must be significantly higher than the concentration of peripheral macrophages already in the tissue. Recalling that in steady state $\hat {M}=0.047$ g/ml, we take *M*_0_=0.05 g/ml. The parameter *α* was estimated by 5, in order to make the asymptotic behavior of $\hat {M}$ in the simulations agree with its assumed steady state of 0.047 g/ml (under Eq. (2)). When microglia cells are activated, they become either of *M*_1_ or *M*_2_ phenotype. But peripheral macrophages are initially biased toward $\hat {M}_{1}$ phenotype rather than $\hat {M}_{2}$ phenotype, since $K_{T_{\alpha }}>K_{I_{10}}$. We assume, in line with this bias toward $\hat {M}_{1}$, that the transition rate from $\hat {M}_{1}$ into $\hat {M}_{2}$ phenotype by TGF- *β* is at a smaller rate than the corresponding transition rate for microglias, that is, $\lambda _{\hat {M}_{1}T_{\beta }}<\lambda _{M_{1}T_{\beta }}$. We take $\lambda _{\hat {M}_{1}T_{\beta }}=6\times 10^{-4}$/day.

#### Eq. (17)

Activated alveolar macrophages produce TNF- *α* at rate 4.86×10^−3^/day [47]. We assume that proinflammatory macrophages produce TNF- *α* at a larger rate (five fold), taking $\lambda _{T_{\alpha } M_{1}}=\lambda _{T_{\alpha } \hat {M}_{1}}=3\times 10^{-2}$ g/ml.

#### Eq. (18)

Astrocytes secrete MCP-1 [17–19] but activated anti-inflammatory microglias also secrete MCP-1. We assume that the production rate by astrocytes in larger than that by *M*_2_, and take $\lambda _{PA}=\frac {1}{2}\lambda _{PM_{2}}$. MCP-1 concentration in initial stages of AD is 750 pg/ml [98]. Using the steady state equation

$$\lambda_{PM_{2}}\frac{1}{2}A_{0}+\lambda_{PM_{2}}M_{2}=d_{P}P, $$

with *P*=6×10^−9^ g/ml and *d*_*P*_=1.73/day [74], we get $\lambda _{PM_{2}}=1.2\times 10^{-7}$/day and *λ*_*PA*_=6×10^−8^/day [47].

Since *A* is increasing in time, also *P* is increasing in time. Hence the steady state assumption needs to be revised. We do it by increasing *λ*_*PA*_ and $\lambda _{PM_{2}}$ by a factor 1.1, taking $\lambda _{PM_{2}}=1.32\times 10^{-7}$/day, and *λ*_*PA*_=6.6×10^−8^/day.

## References

[1] Gatz M, Reynolds CA, Fratiglioni L, Johansson B, Mortimer JA, Berg S, Fiske A, Pedersen NL (2006). Role of genes and environments for explaining Alzheimer disease. Arch Gen Psychiat.

[2] Wilson RS, Barral S, Lee JH, Leurgans SE, Foroud TM, Sweet RA, Graff-Radford N, Bird TD, Mayeux R, Bennett DA (2011). Heritability of different forms of memory in the Late Onset Alzheimer’s Disease Family Study. J Alzheimers Dis..

[3] alzheimers, n.: 2015 Alzheimer’s Statistics. 2016. http://www.alzheimers.net/resources/alzheimers-statistics/. Accessed 1 Sept 2016.

[4] Liu Z, Li P, Wu J, Yi W, Ping L, Xinxin H, et al.The Cascade of Oxidative Stress and Tau Protein Autophagic Dysfunction in Alzheimer’s Disease. Alzheimer’s Dis Challenges Future. 2015;2. doi:10.5772/59980.

[5] Seeman P, Seeman N (2011). Alzheimer’s disease: beta-amyloid plaque formation in human brain. Synapse.

[6] Kremer A, Louis JV, Jaworski T, Van Leuven F (2011). GSK3 and Alzheimer’s Disease: Facts and Fiction. Front Mol Neurosci.

[7] Bloom GS (2014). Amyloid-beta and tau: the trigger and bullet in Alzheimer disease pathogenesis. JAMA Neurol.

[8] Mondragon-Rodriguez S, Perry G, Zhu X, Boehm J (2012). Amyloid Beta and tau proteins as therapeutic targets for Alzheimer’s disease treatment: rethinking the current strategy. Int J Alzheimers Dis.

[9] Wray S, Noble W (2009). Linking amyloid and tau pathology in Alzheimer’s disease: the role of membrane cholesterol in Abeta-mediated tau toxicity. J Neurosci..

[10] Mokhtar SH, Bakhuraysah MM, Cram DS, Petratos S (2013). The Beta-amyloid protein of Alzheimer’s disease: communication breakdown by modifying the neuronal cytoskeleton. Int J Alzheimers Dis.

[11] Joshi P, Turola E, Ruiz A, Bergami A, Libera DD, Benussi L, et. al (2014). Microglia convert aggregated amyloid-beta into neurotoxic forms through the shedding of microvesicles. Cell Death Differ..

[12] Theriault P, ElAli A, Rivest S (2015). The dynamics of monocytes and microglia in Alzheimer’s disease. Alzheimers Res Ther.

[13] de Calignon A, Polydoro M, Suarez-Calvet M, William C, Adamowicz DH, Kopeikina KJ (2012). Propagation of tau pathology in a model of early Alzheimer’s disease. Neuron.

[14] Garwood CJ, Pooler AM, Atherton J, Hanger DP, Noble W (2011). Astrocytes are important mediators of Abeta-induced neurotoxicity and tau phosphorylation in primary culture. Cell Death Dis.

[15] Morales I, Guzman-Martinez L, Cerda-Troncoso C, Farias GA, Maccioni RB (2014). Neuroinflammation in the pathogenesis of Alzheimer’s disease. A rational framework for the search of novel therapeutic approaches. Front Cell Neurosci.

[16] Zhao J, O’Connor T, Vassar R (2011). The contribution of activated astrocytes to A beta production: implications for Alzheimer’s disease pathogenesis. J Neuroinflammation.

[17] Hohsfield LA, Humpel C (2015). Migration of blood cells to beta-amyloid plaques in Alzheimer’s disease. Exp Gerontol..

[18] Li C, Zhao R, Gao K, Wei Z, Yin MY, Lau LT, Chui D, Yu AC (2011). Astrocytes: implications for neuroinflammatory pathogenesis of Alzheimer’s disease. Curr Alzheimer Res.

[19] Porcellini E, Ianni M, Carbone I, Franceschi M, Licastro F (2013). Monocyte chemoattractant protein-1 promoter polymorphism and plasma levels in alzheimer’s disease. Immun Ageing.

[20] Wang WY, Tan MS, Yu JT, Tan L (2015). Role of pro-inflammatory cytokines released from microglia in Alzheimer’s disease. Ann Transl Med.

[21] Lai AY, McLaurin J (2012). Clearance of amyloid-beta peptides by microglia and macrophages: the issue of what, when and where. Future Neurol.

[22] Bhaskar K, Maphis N, Xu G, Varvel NH, Kokiko-Cochran ON, Weick JP (2014). et al. Microglial derived tumor necrosis factor-alpha drives Alzheimer’s disease-related neuronal cell cycle events. Neurobiol Dis..

[23] Sharma V, Thakur V, Singh S, Guleria R (2012). Tumor Necrosis Factor and Alzheimer’s Disease: A Cause and Consequence Relationship. Klinik Psik Bull Clin Psyc..

[24] Boutajangout A, Sigurdsson EM, Krishnamurthy PK (2011). Tau as a therapeutic target for Alzheimer’s disease. Curr Alzheimer Res.

[25] Hong-Qi Y, Zhi-Kun S, Sheng-Di C (2012). Current advances in the treatment of Alzheimer’s disease: focused on considerations targeting Abeta and tau. Transl Neurodegener.

[26] Lansdall C. An effective treatment for Alzheimer’s disease must consider both amyloid and tau. Biosci Horizons. 2014;7. doi:10.1093/biohorizons/hzu002.

[27] Wischik CM, Harrington CR, Storey JM (2014). Tau-aggregation inhibitor therapy for Alzheimer’s disease. Biochem Pharmacol..

[28] Lee MH, Lin SR, Chang JY, Schultz L, Heath J, Hsu LJ, Kuo YM, Hong Q, Chiang MF, Gong CX, Sze CI, Chang NS (2010). TGF-beta induces TIAF1 self-aggregation via type II receptor-independent signaling that leads to generation of amyloid beta plaques in Alzheimer’s disease. Cell Death Dis.

[29] Chao CC, Hu S, Frey WH, Ala TA, Tourtellotte WW, Peterson PK (1994). Transforming growth factor beta in Alzheimer’s disease. Clin Diagn Lab Immunol..

[30] Chen JH, Ke KF, Lu JH, Qiu YH, Peng YP (2015). Protection of TGF-beta against neuroinflammation and neurodegeneration in Abeta1–42-induced Alzheimer’s disease model rats. PLoS ONE.

[31] Das P, Golde T (2006). Dysfunction of TGF-beta signaling in Alzheimer’s disease. J Clin Invest..

[32] von Bernhardi R, Cornejo F, Parada GE, Eugenin J (2015). Role of TGF beta signaling in the pathogenesis of Alzheimer’s disease. Front Cell Neurosci.

[33] Wyss-Coray T (2006). Tgf-Beta pathway as a potential target in neurodegeneration and Alzheimer’s. Curr Alzheimer Res.

[34] Wyss-Coray T, Lin C, Yan F, Yu GQ, Rohde M, McConlogue L, Masliah E, Mucke L (2001). TGF-beta1 promotes microglial amyloid-beta clearance and reduces plaque burden in transgenic mice. Nat Med..

[35] Town T, Laouar Y, Pittenger C, Mori T, Szekely CA, Tan J (2008). Blocking TGF-beta-Smad2/3 innate immune signaling mitigates Alzheimer-like pathology. Nat Med..

[36] Cochran JN, Hall AM, Roberson ED (2014). The dendritic hypothesis for Alzheimer’s disease pathophysiology. Brain Res Bull..

[37] Dorostkar MM, Zou C, Blazquez-Llorca L, Herms J (2015). Analyzing dendritic spine pathology in Alzheimer’s disease: problems and opportunities. Acta Neuropathol..

[38] Klyubin I, Cullen WK, Hu NW, Rowan MJ (2012). Alzheimer’s disease Abeta assemblies mediating rapid disruption of synaptic plasticity and memory. Mol Brain.

[39] Koffie RM, Hyman BT, Spires-Jones TL (2011). Alzheimer’s disease: synapses gone cold. Mol Neurodegener.

[40] Craft DL, Wein LM, Selkoe DJ (2002). A mathematical model of the impact of novel treatments on the A beta burden in the Alzheimer’s brain, CSF and plasma. Bull Math Biol..

[41] Bertsch M, Franchi B, Marcello N, Tesi MC, Tosin A. Alzheimer’s disease: a mathematical model for onset and progression. Math Med Biol. 2016. doi:10.1093/imammb/dqw003. 10.1093/imammb/dqw003 27079222

[42] Helal M, Hingant E, Pujo-Menjouet L, Webb GF (2014). Alzheimer’s disease: analysis of a mathematical model incorporating the role of prions. J Math Biol.

[43] Puri IK, Li L (2010). Mathematical modeling for the pathogenesis of Alzheimer’s disease. PLoS ONE.

[44] Lao A, Schmidt V, Schmitz Y, Willnow TE, Wolkenhauer O (2012). Multi-compartmental modeling of SORLA’s influence on amyloidogenic processing in Alzheimer’s disease. BMC Syst Biol.

[45] Schmidt V, Baum K, Lao A, Rateitschak K, Schmitz Y, Teichmann A (2012). Quantitative modelling of amyloidogenic processing and its influence by SORLA in Alzheimer’s disease. EMBO J..

[46] Hamza T, Barnett JB, Li B (2010). Interleukin 12 a key immunoregulatory cytokine in infection applications. Int J Mol Sci.

[47] Hao W, Crouser ED, Friedman A (2014). Mathematical model of sarcoidosis. Proc Nat Acad Sci USA.

[48] Sokolowski JD, Mandell JW (2011). Phagocytic clearance in neurodegeneration. Am J Pathol..

[49] Haass C, Selkoe DJ (2007). Soluble protein oligomers in neurodegeneration: lessons from the Alzheimer’s amyloid beta-peptide. Nat Rev Mol Cell Biol..

[50] Waters J (2010). The concentration of soluble extracellular amyloid-beta protein in acute brain slices from CRND8 mice. PLoS ONE.

[51] Muller S, Ronfani L, Bianchi ME (2004). Regulated expression and subcellular localization of HMGB1, a chromatin protein with a cytokine function. J Intern Med..

[52] Gao HM, Zhou H, Zhang F, Wilson BC, Kam W, Hong JS (2011). HMGB1 acts on microglia Mac1 to mediate chronic neuroinflammation that drives progressive neurodegeneration. J Neurosci..

[53] Lotze MT, Tracey KJ (2005). High-mobility group box 1 protein (HMGB1): nuclear weapon in the immune arsenal. Nat Rev Immunol..

[54] Zou JY, Crews FT (2014). Release of neuronal HMGB1 by ethanol through decreased HDAC activity activates brain neuroimmune signaling. PLoS ONE.

[55] Savchenko VL, McKanna JA, Nikonenko IR, Skibo GG (2000). Microglia and astrocytes in the adult rat brain: comparative immunocytochemical analysis demonstrates the efficacy of lipocortin 1 immunoreactivity. Neuroscience.

[56] Hao W, Rovin BH, Friedman A (2014). Mathematical model of renal interstitial fibrosis. Proc Nat Acad Sci USA.

[57] Roher AE, Esh CL, Kokjohn TA, Castano EM, Van Vickle GD, Kalback WM (2009). Amyloid beta peptides in human plasma and tissues and their significance for Alzheimer’s disease. Alzheimers Dement.

[58] Furman JL, Sama DM, Gant JC, Beckett TL, Murphy MP, Bachstetter AD, Van Eldik LJ, Norris CM (2012). Targeting astrocytes ameliorates neurologic changes in a mouse model of Alzheimer’s disease. J Neurosci..

[59] Tobinick E, Gross H, Weinberger A, Cohen H (2006). TNF-alpha modulation for treatment of Alzheimer’s disease: a 6-month pilot study. MedGenMed.

[60] Butchart J, Brook L, Hopkins V, Teeling J, Puntener U, Culliford D (2015). Etanercept in Alzheimer disease: A randomized, placebo-controlled, double-blind, phase 2 trial. Neurology.

[61] Piazza F, Winblad B (2016). Amyloid-Related Imaging Abnormalities (ARIA) in Immunotherapy Trials for Alzheimer’s Disease: Need for Prognostic Biomarkers?. J Alzheimers Dis..

[62] Karran E, Hardy J (2014). A critique of the drug discovery and phase 3 clinical programs targeting the amyloid hypothesis for Alzheimer disease. Ann Neurol..

[63] Patel KR (2015). Biogen’s aducanumab raises hope that Alzheimer’s can be treated at its source. Manag Care.

[64] Reardon S (2015). Antibody drugs for Alzheimer’s show glimmers of promise. Nature.

[65] Ge S, Shrestha B, Paul D, Keating C, Cone R, Guglielmotti A, Pachter JS (2012). The CCL2 synthesis inhibitor bindarit targets cells of the neurovascular unit, and suppresses experimental autoimmune encephalomyelitis. J Neuroinflammation.

[66] Severini C, Passeri PP, Ciotti M, Florenzano F, Possenti R, Zona C (2014). Bindarit, inhibitor of CCL2 synthesis, protects neurons against amyloid-??-induced toxicity. J Alzheimers Dis..

[67] Li JY, Ren YP, Yuan Y, Ji SM, Zhou SP, Wang LJ, Mou ZZ, Li L, Lu W, Zhou TY (2016). Preclinical PK/PD model for combined administration of erlotinib and sunitinib in the treatment of A549 human NSCLC xenograft mice. Acta Pharmacol Sin..

[68] Nielsen EI, Cars O, Friberg LE (2011). Pharmacokinetic/pharmacodynamic (PK/PD) indices of antibiotics predicted by a semimechanistic PKPD model: a step toward model-based dose optimization. Antimicrob Agents Chemother..

[69] Yuan Y, Zhou X, Ren Y, Zhou S, Wang L, Ji S, Hua M, Li L, Lu W, Zhou T (2015). Semi-Mechanism-Based Pharmacokinetic/Pharmacodynamic Model for the Combination Use of Dexamethasone and Gemcitabine in Breast Cancer. J Pharm Sci.

[70] Bloom GS (2014). Amyloid-beta and tau: the trigger and bullet in Alzheimer disease pathogenesis. JAMA Neurol.

[71] Marino S, Hogue IB, Ray CJ, Kirschner DE (2008). A methodology for performing global uncertainty and sensitivity analysis in systems biology. J Theor Biol..

[72] Collins-Praino LE, Francis YI, Griffith EY, Wiegman AF, Urbach J, Lawton A, Honig LS, Cortes E, Vonsattel JP, Canoll PD, Goldman JE, Brickman AM (2014). Soluble amyloid beta levels are elevated in the white matter of Alzheimer’s patients, independent of cortical plaque severity. Acta Neuropathol Commun.

[73] Mohs RC, Haroutunian V (1999). Chapter 82: Alzheimer Disease: From Earliest Symptoms to End Stage. Neuropsychopharmacology: The Fifth Generation of Progress.

[74] Hao W, Friedman A (2014). The LDL-HDL profile determines the risk of atherosclerosis: a mathematical model. PLoS ONE.

[75] Young ME, Carroad PA, Bell RL (1980). Estimation of Diffusion Coefficients of Proteins. Biot Bioe.

[76] Chen D, Roda JM, Marsh CB, Eubank TD, Friedman A (2012). Hypoxia inducible factors-mediated inhibition of cancer by GM-CSF: a mathematical model. Bull Math Biol..

[77] Yokochi S, Hashimoto H, Ishiwata Y, Shimokawa H, Haino M, Terashima Y, Matsushima K (2001). An anti-inflammatory drug, propagermanium, may target GPI-anchored proteins associated with an MCP-1 receptor, CCR2. J Interferon Cytokine Res..

[78] Dubois CM, Laprise MH, Blanchette F, Gentry LE, Leduc R (1995). Processing of transforming growth factor beta 1 precursor by human furin convertase. J Biol Chem..

[79] Stepanets OV, Chichasova NV, Nasonova MB, Samsonov MIU, Nasonov EL (2003). [Soluble receptors of TNF-alpha with molecular mass 55 kDa in rheumatoid arthritis: clinical role]. Klin Med (Mosk).

[80] Bonaldi T, Talamo F, Scaffidi P, Ferrera D, Porto A, Bachi A, Rubartelli A, Agresti A, Bianchi ME (2003). Monocytic cells hyperacetylate chromatin protein HMGB1 to redirect it towards secretion. EMBO J..

[81] Ahmed M, Davis J, Aucoin D, Sato T, Ahuja S, Aimoto S, Elliott JI, Van Nostrand WE, Smith SO (2010). Structural conversion of neurotoxic amyloid-beta(1–42) oligomers to fibrils. Nat Struct Mol Biol..

[82] Saido T, Leissring MA (2012). Proteolytic degradation of amyloid beta-protein. Cold Spring Harb Perspect Med.

[83] Cragg BG (1975). The density of synapses and neurons in normal, mentally defective ageing human brains. Brain.

[84] Gate D, Rezai-Zadeh K, Jodry D, Rentsendorj A, Town T (2010). Macrophages in Alzheimer’s disease: the blood-borne identity. J Neural Transm (Vienna).

[85] Heppner FL, Ransohoff RM, Becher B (2015). Immune attack: the role of inflammation in Alzheimer disease. Nat Rev Neurosci..

[86] Herculano-Houzel S (2009). The human brain in numbers: a linearly scaled-up primate brain. Front Hum Neurosci.

[87] Herculano-Houzel S (2014). The glia/neuron ratio: how it varies uniformly across brain structures and species and what that means for brain physiology and evolution. Glia.

[88] Poppek D, Keck S, Ermak G, Jung T, Stolzing A, Ullrich O, Davies KJ, Grune T (2006). Phosphorylation inhibits turnover of the tau protein by the proteasome: influence of RCAN1 and oxidative stress. Biochem J..

[89] Kapaki E, Kilidireas K, Paraskevas GP, Michalopoulou M, Patsouris E (2001). Highly increased CSF tau protein and decreased beta-amyloid (1–42) in sporadic CJD: a discrimination from Alzheimer’s disease?. J Neurol Neurosurg Psychiatr..

[90] Hao W, Schlesinger LS, Friedman A (2016). Modeling Granulomas in Response to Infection in the Lung. PLoS ONE.

[91] Elson K, Ribeiro RM, Perelson AS, Simmons A, Speck P (2004). The life span of ganglionic glia in murine sensory ganglia estimated by uptake of bromodeoxyuridine. Exp Neurol..

[92] Maree AF, Komba M, Finegood DT, Edelstein-Keshet L (2008). A quantitative comparison of rates of phagocytosis and digestion of apoptotic cells by macrophages from normal (BALB/c) and diabetes-prone (NOD) mice. J Appl Physiol..

[93] Wang J, Dickson DW, Trojanowski JQ, Lee VM (1999). The levels of soluble versus insoluble brain Abeta distinguish Alzheimer’s disease from normal and pathologic aging. Exp Neurol..

[94] Zhu XD, Chen JS, Zhou F, Liu QC, Chen G, Zhang JM (2012). Relationship between plasma high mobility group box-1 protein levels and clinical outcomes of aneurysmal subarachnoid hemorrhage. J Neuroinflammation.

[95] Allette YM, Due MR, Wilson SM, Feldman P, Ripsch MS, Khanna R, White FA (2014). Identification of a functional interaction of HMGB1 with Receptor for Advanced Glycation End-products in a model of neuropathic pain. Brain Behav Immun..

[96] Rezai-Zadeh K, Gate D, Gowing G, Town T (2011). How to get from here to there: macrophage recruitment in Alzheimer’s disease. Curr Alzheimer Res.

[97] Rezai-Zadeh K, Gate D, Town T (2009). CNS infiltration of peripheral immune cells: D-Day for neurodegenerative disease?. J Neuroimmune Pharmacol.

[98] Westin K, Buchhave P, Nielsen H, Minthon L, Janciauskiene S, Hansson O (2012). CCL2 is associated with a faster rate of cognitive decline during early stages of Alzheimer’s disease. PLoS ONE.

[99] Hao W, Marsh C, Friedman A (2015). A Mathematical Model of Idiopathic Pulmonary Fibrosis. PLoS ONE.

